# Tumor microenvironment restricts IL-10 induced multipotent progenitors to myeloid-lymphatic phenotype

**DOI:** 10.1371/journal.pone.0298465

**Published:** 2024-04-19

**Authors:** Lisa Volk-Draper, Shaswati Athaiya, Maria Espinosa Gonzalez, Nihit Bhattarai, Andrew Wilber, Sophia Ran

**Affiliations:** 1 Department of Medical Microbiology, Immunology, and Cell Biology, Southern Illinois University School of Medicine, Springfield, IL, United States of America; 2 Simmons Cancer Institute, Southern Illinois University School of Medicine, Springfield, IL, United States of America; CCAC, UNITED STATES

## Abstract

Lymphangiogenesis is induced by local pro-lymphatic growth factors and bone marrow (BM)-derived myeloid-lymphatic endothelial cell progenitors (M-LECP). We previously showed that M-LECP play a significant role in lymphangiogenesis and lymph node metastasis in clinical breast cancer (BC) and experimental BC models. We also showed that differentiation of mouse and human M-LECP can be induced through sequential activation of colony stimulating factor-1 (CSF-1) and Toll-like receptor-4 (TLR4) pathways. This treatment activates the autocrine interleukin-10 (IL-10) pathway that, in turn, induces myeloid immunosuppressive M2 phenotype along with lymphatic-specific proteins. Because IL-10 is implicated in differentiation of numerous lineages, we sought to determine whether this pathway specifically promotes the lymphatic phenotype or multipotent progenitors that can give rise to M-LECP among other lineages. Analyses of BM cells activated either by CSF-1/TLR4 ligands *in vitro* or orthotopic breast tumors *in vivo* showed expansion of stem/progenitor population and coincident upregulation of markers for at least four lineages including M2-macrophage, lymphatic endothelial, erythroid, and T-cells. Induction of cell plasticity and multipotency was IL-10 dependent as indicated by significant reduction of stem cell markers and those for multiple lineages in differentiated cells treated with anti-IL-10 receptor (IL-10R) antibody or derived from IL-10R knockout mice. However, multipotent CD11b^+^/Lyve-1^+^/Ter-119^+^/CD3e^+^ progenitors detected in BM appeared to split into a predominant myeloid-lymphatic fraction and minor subsets expressing erythroid and T-cell markers upon establishing tumor residence. Each sub-population was detected at a distinct intratumoral site. This study provides direct evidence for differences in maturation status between the BM progenitors and those reaching tumor destination. The study results suggest preferential tumor bias towards expansion of myeloid-lymphatic cells while underscoring the role of IL-10 in early BM production of multipotent progenitors that give rise to both hematopoietic and endothelial lineages.

## Introduction

The lymphatic system plays vital roles in metabolism, homeostasis, and immunity. The main functions of the lymphatic system are absorption of interstitial fluid and proteins, transport of immune cells as well as regulation of inflammatory and immune responses [1, 2]. Functional insufficiency of the lymphatic system is the underlying cause of primary and secondary lymphedema [2] whereas excessive formation of tumor lymphatic vessels promotes metastasis [3, 4]. Increase in lymphatic vessel density (LVD) at chronically inflamed sites also has significant implications for regulation of metabolic and immune disorders [5]. Previously published studies presented substantial evidence for critical effects of expansion of the lymphatic vasculature on outcomes of chronic inflammatory diseases and cancers. Advancing mechanistic understanding of the formation of new lymphatic vessels (i.e., lymphangiogenesis) can promote development of corrective treatments for patients with major human diseases who currently have limited therapeutic options.

Lymphangiogenesis is thought to be induced by soluble lymphatic-promoting factors, bone marrow (BM)-derived lymphatic progenitors, or a combination of both. The canonical mechanism suggests that lymphangiogenesis is driven primarily by binding vascular endothelial growth factor-C (VEGF-C) or similar factor VEGF-D to their receptor VEGFR-3 on lymphatic endothelial cells (LEC) [6, 7]. Indeed, VEGFR-3 activation is known to increase division, migration, and tube formation of LEC *in vitro* and the formation of new lymphatic sprouts *in vivo* [6–8]. However, it has also been proposed that lymphatic sprouting is mediated by BM-derived myeloid-lymphatic endothelial cell progenitors (M-LECP) whose differentiation relies, in part, on VEGFR-3 signaling [9]. This mechanism is supported by multiple lines of evidence. M-LECP, identified by their unique profile of stem-myeloid-lymphatic markers, have been detected in multiple inflammatory and tumor models including gastric [10], colorectal [11], breast [12] and other tissue-specific cancers [13]. Blood circulating and tumor-recruited M-LECP have been detected in human patients with breast and lung malignancies [14–16]. The levels of mouse and human M-LECP strongly correlate with increased tumor LVD and lymphatic metastasis [12, 16]. Lymphatic progenitors can promote vessel formation through several mechanisms including increased production of VEGF-C [14] and other lymphangiogenic factors [17], integration into tumor lymphatic vessels [18–20] and donation of their intracellular contents to tumor LEC [13]. Although the precise mechanisms underlying M-LECP dependent lymphangiogenesis are still under debate, significant correlations of their blood and tumor levels with LVD and node metastasis in human cancer patients [14–16] provide a strong impetus to characterize their BM origin, differentiation, and lineage fate upon recruitment to tumors.

Our prior analysis of surface markers of M-LECP in clinical breast cancer (BC) showed that they share characteristics of tumor-associated macrophages (TAMs) of the immunosuppressive M2-type [16] that typically dominate immune infiltrates in human cancers [21]. Polarization to M2 macrophages is regulated by Th2 cytokines IL-10 [22], IL-4 [23] and IL-13 [24]. We recently established that exogenous addition of any of the Th2 factors promotes M-LECP differentiation, but only IL-10 and IL-10 receptor (IL-10R) are co-expressed during this process [25]. Receptors for IL-4 and IL-13 were upregulated to the same level as IL-10R but unlike IL-10, BM cells expressed no corresponding ligands [25]. While IL-10 alone could be responsible for both M2 and lymphatic phenotypes of tumor M-LECP [16], it raised the question of specificity of its effect as this pathway is also involved in determining the fate of many other lineages [26–28].

In addition to advancing differentiation of both blood vascular [29, 30] and lymphatic endothelial cell precursors [25], IL-10 promotes generation of at least nine other cell lineages including monocytes [27, 31], M2 macrophages [32], B-cells [28, 33], T-cells [34], osteoblasts [35, 36], chondrocytes [37, 38], adipocytes [39], intestinal epithelial cells [40], and oligodendrocytes [41]. This effect of IL-10 has been shown in human and mouse immature precursors [36, 38] that were isolated, respectively, from blood of patients with inflammatory disorders [36, 42, 43] and mouse BM [26]. The impact of IL-10 pathway on multiple lineages might be due, in part, to its pro-survival and pro-mitotic effects on multipotent hematopoietic [26] and mesenchymal stem cells (MSC) [44] located at the apex of their respective hierarchical trees. Hematopoietic stem cells (HSC) from IL-10 knockout (KO) mice failed to reconstitute lethally irradiated wild-type (WT) mice demonstrating IL-10 dependency of downstream generation of immune/endothelial lineages necessary for homeostasis and regeneration [26]. Consistently, over-expression of IL-10 in mouse injury models significantly increased engraftment of MSC that facilitate tissue repair [44]. The plurality of IL-10 effects on cell differentiation suggested its control of the top elements of the developmental hierarchy rather than promotion of specific lineages. This concept led us to hypothesize that BM-derived IL-10 confers the multipotent potential to early myeloid precursors that might undergo subsequent pro-lymphatic specialization after release from the BM in tumors or other extramedullar site.

To test this hypothesis, we quantified expression of stemness indicators as well as hematopoietic and endothelial markers in BM-derived M-LECP differentiated *in vitro* [12] or induced in BM by orthotopic breast tumors *in vivo*. We found that exposure of CSF-1 primed BM cells to IL-10 led to generation of M2-polarized multipotent progenitors identified by co-expression of specific markers for stem cells as well as myeloid, lympho-endothelial, T-cell lymphoid, and erythroid lineages. In contrast, M-LECP residing in established human or mouse breast tumors *in vivo* lacked T-cell and erythroid markers while retaining the myeloid-lymphatic phenotype. Consistently, subsets expressing T-cell and erythroid markers lost a lymphatic marker Lyve-1 and were physically segregated from M-LECP by migrating to independent tumor locales. These novel findings suggest that IL-10 in the BM drives generation of immature regenerative cells with diverse lineage potential but tumor-residing progenitors have advanced maturity acquired either at an extramedullar site or in response to tumor-specific cues. This further suggests that tumor microenvironment (TME) can influence cell composition and the terminal lineage fate of recruited progenitors.

## Materials and methods

### Materials

Dulbecco’s low glucose modified Eagle’s medium (DMEM) without phenol red, standard medium supplements, TrypLE, human plasma fibronectin, and Dulbecco’s phosphate buffered saline (DPBS), Image-iT FX signal enhancer, Hoechst stain, and Prolong Gold medium were purchased from Thermo Scientific (Rockford, IL). Fetal bovine serum (FBS) was purchased from Biowest USA (Bradenton, FL). Mouse gamma globulins and LPS from *E*. *coli* 055:B5 were obtained from Millipore Sigma (St. Louis, MO). Paraformaldehyde was from Electron Microscopy Sciences (Hatfield, PA). CSF-1 was from BioLegend (San Diego, CA, cat#576406).

### Animal ethics statement

Animal experiments were conducted in accordance with recommendations cited in the Guide for the Care and Use of Laboratory Animals of the National Institute of Health. Protocols #187-13-021 and #187-20-004 were approved by the Laboratory Animal Care and Use Committee of Southern Illinois University School of Medicine. All considerations were taken to minimize pain and distress. In this study, 50 mice were used for experiments that lasted no longer than 6 weeks. All mice were euthanized at planned experimental endpoints.

### Antibodies

All antibodies used for flow cytometry and immunofluorescence are listed in S1 Table. Rat anti-mouse monoclonal blocking IL-10R antibody (cat# BE0050) and isotype-matched control rat IgG of irrelevant specificity (cat#BE0290) were purchased from BioXcell (Lebanon, NH).

### Isolation and differentiation of BM cells from wild-type (WT) and IL-10R knockout (KO) mice

BM cells isolated from female C57BL/6J (WT) and B6.129S2-IL10rb^tm/AgtJ^ (IL10R-KO) mice (Jackson Labs, Bar Harbor, ME) were used in an *in vitro* differentiation assay. Mice were placed in a bell jar with isoflurane and euthanized by inhalation overdose. One minute after the final respiration, a cervical dislocation was performed and long bones removed. BM cells obtained from long bones were crushed using a mortar and pestle in 10 mL of DPBS containing 0.5% BSA and 2 mM EDTA. Cell suspension was passed through a 70-μm strainer and pelleted at 1,000 rpm for 10 minutes. Standard growth medium was DMEM containing 10% FBS and standard growth supplements. Cells were seeded at the density of 10x10^6^ cells in 10 mL of growth medium containing 10 ng/mL of CSF-1 on 10-cm-diameter dishes precoated with 10 μg/mL of fibronectin. After three days, medium and nonadherent cells were removed. The remaining attached cells were washed three times with DPBS, and stimulated with of CSF-1 (10 ng/mL) and LPS (3 nM) in the presence of 5 μg/mL isotype control or anti-IL-10R IgG for three additional days. GFP-tagged M-LECP were produced using an identical protocol from BM cells of C57BL6-Tg(UBC-GFP)30SchaJ mice (Jackson Labs) that have ubiquitous GFP expression under transcriptional control of the Ubiquitin C promoter. On day 6, cells were analyzed by flow cytometry or RT-qPCR using antibodies and primers listed in S1 and S2 Tables, respectively.

### Flow cytometry analysis of single and multicolor staining

Flow cytometry was performed using 2x10^5^ cells per sample. Fc receptor was blocked by a 10-minute incubation on ice with 10 μg/mL of mouse gamma globulins. Blocked cells were incubated with 5 μg/mL of primary antibodies for one hour on ice, washed with ice-cold F-buffer (2% BSA and 0.2% of sodium azide in DPBS), and incubated with 1 μg/mL of 488-conjugated secondary antibody for one hour on ice followed by washing with F-buffer. For multicolor analyses, cells with blocked Fc receptors were co-stained with primary antibodies against markers of lymphatic (Lyve-1, Pdpn), myeloid (CD11b, TLR4) or hematopoietic lineages. After a 1-hour incubation on ice and washing, cells were incubated with appropriate 488-, or 647-conjugated secondary antibodies. After a 30-minute incubation on ice, cells were fixed for 10 minutes with 1% paraformaldehyde, washed, and resuspended in 250 μL of F-buffer. Targets were detected using an Accuri™ C6 and FACS ARIA II flow cytometer (BD Accuri Cytometers, San Jose, CA) for single and double staining, respectively. Data analyzed by FlowJo software (Tree Star, OR) are presented as histograms or dot plots. The gating for all primary antibodies was determined based on negative staining established by an appropriate secondary antibody alone. In some cases, myeloid CD11b or TLR4 positive cells were gated from the total population and percentage of cells with co-expression of lymphatic or other markers determined in duplicate for each sample. Results are presented as the mean percentage of positive cells ± S.D. All experiments were reproduced at least twice.

### RT-qPCR analysis

RNA was extracted using RNAeasy columns (Qiagen, Caldwell, NJ) and reverse transcribed with SuperScript VILO cDNA synthesis kit (Thermo Fisher). Primers were designed based on published coding sequences (CDS) of mouse targets found in NCBI database. All primer sequences are listed in S2 Table. RT-qPCR was performed using GoTaq Green Master Mix (Promega, Madison, WI) and MasterCycle Realplex PCR machine (Eppendorf, Hamburg, Germany). Data were normalized to β-actin and are presented as fold-change in target expression in WT *ex-vivo* cells compared to LPS differentiated cells, WT cells differentiated in the presence of anti-IL-10R antibody relative to control IgG, or in differentiated WT cells relative to identically treated BM cells from IL-10R knockout mice.

### Culture of human and mouse breast carcinoma cell lines

Human breast carcinoma ZR-75 and mouse EMT6 cell lines were obtained from ATCC (Manassas, VA). Cell lines were cultured in DMEM containing 5% FBS, and standard supplements at 37°C in 10% CO_2_. Cells were passaged biweekly by incubating for five minutes at 37°C in 0.5mM of EDTA in DPBS followed by TrypLE. Cells were routinely tested for mycoplasma by e-Myco Mycoplasma PCR Detection Kit (Bulldog Bio, Portsmouth, NH). ZR-75 line was authenticated by ATCC and EMT6 line was screened by Impact III testing through RADIL (Columbia, MO) for mouse pathogens and determined to be negative.

### Tumor induction of xenograft and syngeneic orthotopic BC models

Orthotopic implantation and growth pattern of ZR-75 (human) and EMT6 (mouse) breast carcinoma cell lines in SCID and BALB/c mice, respectively, were described previously [12]. Briefly, 5 to 6-week-old CB-17/SCID or BALB/C mice (Taconic, Hudson, NY) were anesthetized with isoflurane followed by the mammary fat pad (MFP) implantation of 4x10^6^ of ZR-75 or 1x10^6^ of EMT6 cells suspended in 50% Matrigel. Every 2–3 days, perpendicular tumor diameters were measured by digital calipers and used to calculate tumor volume according to the formula: volume = Dd^2^π/6, where D and d equal to larger and smaller diameters, respectively. Animal health and behavior was monitored 2–3 times per week along with monitoring the endpoint criteria such as becoming non-responsive to stimuli, hypothermic, losing more than 20% of the initial body weight, distorted posture, respiratory distress, tumor ulceration, or a tumor volume exceeding 1.8 cm^3^. Any mice that would meet such criteria would be euthanized within 24 hours. Mice that met planned experimental endpoints were placed in a bell jar with isoflurane and euthanized by inhalation overdose. One minute after final respiration, mice were perfused and tissues collected for analyses.

### Analyses of exogenous and endogenous M-LECP in ZR-75 and EMT6 tumors *in vivo*

Analyses of tumor-residing M-LECP for co-expression of hematopoietic markers were performed using both *in vitro* and *in vivo* produced progenitors. To monitor recruitment and marker profile of exogenous M-LECP, BM cells from GFP-expressing mice were differentiated using the CSF-1/LPS protocol followed by intravenous injection of 1.5x10^6^ cells into mice with established ZR-75 tumors (~500 mm^3^). The M-LECP signature profile (Sca-1^+^/CD11b^+^/Lyve-1^+^/Pdpn^+^/Ter-119^+^/CD3e^+^) was validated for differentiated GFP^+^ cells by flow cytometry immediately before adoptive transfer. Tumors were harvested upon reaching 1,800 mm^3^ and analyzed for the presence of Lyve-1^+^ cells that either co-expressed or lacked GFP, a method that allowed their classification as exogenous or endogenous progenitors, respectively. Tumor-induced endogenous M-LECP were also analyzed in the mouse syngeneic EMT6 tumors collected on the day 2, 4, 7 and 10 post-implantation (N = 4 per timepoint). Snap-frozen tumors were sectioned and stained with various antibodies to determine recruitment of Lyve-1^+^ cells with and without GFP that co-expressed CD11b, Ter-119 or CD3e markers.

### Immunohistochemistry (IHC) and immunofluorescence

Acetone-fixed 8-μm thick frozen tumor sections were rehydrated in PBS with 0.1% Tween-20 (PBST) before a 30-minute incubation with Image-iT FX signal enhancer at room temperature. Primary antibodies diluted 1:100 in PBST containing 0.2% BSA (IHC buffer) were incubated with sections overnight at 4°C. Slides were washed for 10 minutes in PBST. Secondary antibodies diluted 1:100 in IHC buffer were incubated at 37°C for 1 hour. Slides were washed and incubated for 5 minutes at room temperature with 2 μg/mL of Hoechst dye. Slides were fixed with 1% paraformaldehyde, washed in PBST for 10 minutes, and mounted in Prolong Gold medium. Images were acquired using a Zeiss LSM800 confocal microscope equipped with high-resolution Airyscan and analyzed using Zen Blue 2.6 software (Carl Zeiss GbmH, Jena, Germany).

### Quantification of tumor-recruited Lyve-1^+^ cells co-expressing lineage-specific markers

ZR-75 and EMT6 tumor sections (N = 4–5 per model) were co-stained with antibodies against Lyve-1 and CD11b, CD3e or Ter-119. Exogenous M-LECP were detected by triple staining with the above antibodies and GFP. A minimum of 100 Lyve-1^+^ cells with or without GFP were quantified for co-localization with each of the lineage markers. These data are presented as the mean percent of Lyve-1^+^/GFP-negative or Lyve-1^+^/GFP-positive cells co-expressing lineage-specific markers ± S.D.

### Statistical analysis

Statistical significance of differences among groups was determined by Student’s t-test using GraphPad Prism6 software. A P-value ≤0.05 was considered significant.

## Results

### CSF-1/TLR4 induced differentiation promotes myeloid-endothelial progenitors with predominant lymphatic phenotype

We previously showed that human monocytes and mouse BM cells activated by CSF-1 and TLR4 ligands co-regulate expression of myeloid and lymphatic proteins including Lyve-1 and podoplanin (Pdpn) [9, 12]. We termed this subset myeloid-lymphatic progenitors (M-LECP) because these BM-derived cells have been shown to be recruited to tumors and other remodeling sites and significantly contribute to lymphatic vascular sprouting *in vivo* [12, 13, 16]. This evidence suggests a significant role of M-LECP in promoting tumor lymphatics and metastasis but their properties and differentiation mechanisms remained poorly defined. Here, we expanded characterization of marker profile of M-LECP produced *in vitro* and in BM *in vivo*, and identified IL-10 as one of the regulators of their differentiation as well as several other lineages.

**Fig 1:**
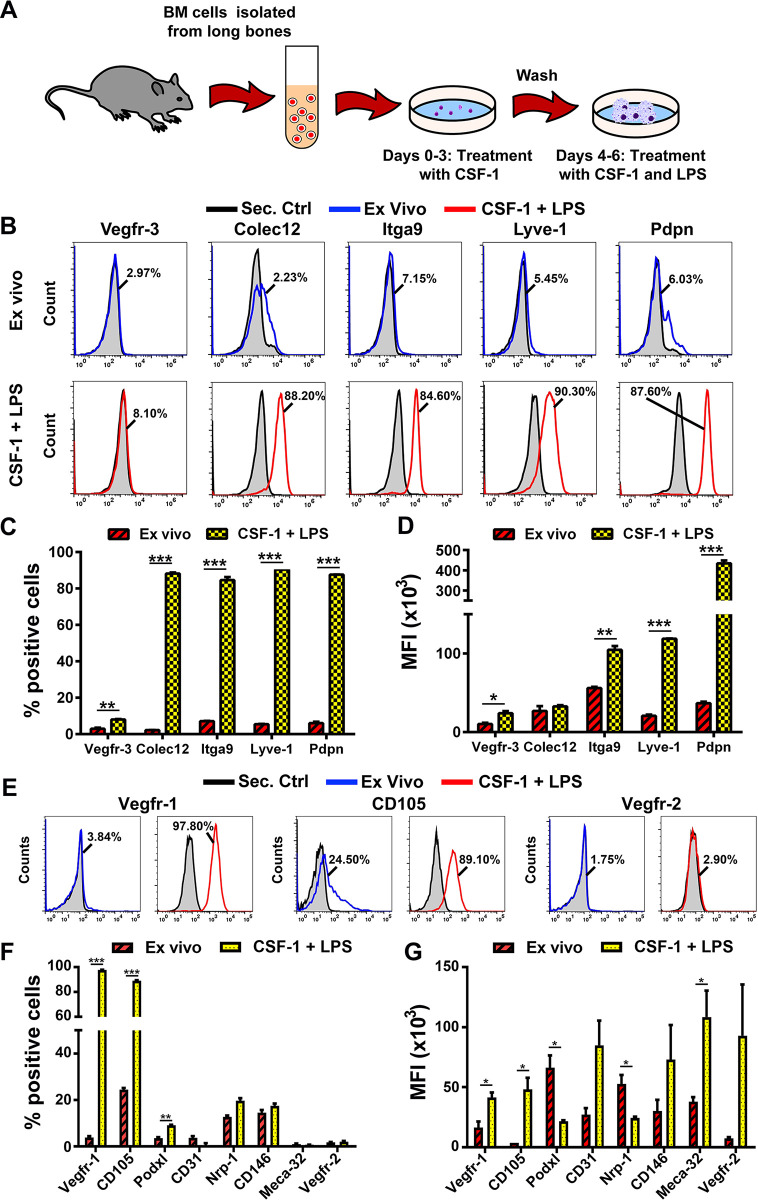
CSF-1/TLR4 induced differentiation promotes endothelial progenitors with predominant lymphatic phenotype. (A) Schematic of the protocol used to differentiate BM cells by sequential activation of CSF-1 and TLR4 pathways. (B) Representative histograms demonstrating expression of lymphatic specific targets Vegfr-3, Colec12, Itga9, Lyve-1, and Pdpn in freshly-isolated BM cells (upper panel, Ex-vivo, blue line) and cells differentiated with CSF-1 and LPS (lower panel, red line). Numbers pointing to blue and red lines indicate the percentage of marker-positive cells. Secondary controls are shown by the grey area outlined by black line in each panel. (C) The mean percentage of positive cells and (D) MFI (x10^3^) of BM cells expressing LEC markers. (E) Representative histograms demonstrating the percentage of Ex-vivo (blue line) and differentiated (red line) cells positive for blood vascular endothelial markers Vegfr-1, CD105, and Vegfr-2. Secondary controls are shown by the grey area outlined by black line in each panel. (F) The mean percentage of positive cells and (G) MFI (x10^3^) of various endothelial markers in *ex-vivo* and differentiated cells. Black bars indicate statistical significance determined by Student’s t-test with P-values shown by *<0.05, **<0.01, and ***<0.001. All experiments were performed in duplicate and reproduced three times.

Fig 1A illustrates our differentiation protocol that consists of CSF-1 priming followed by TLR4 activation applied to naïve BM cells from healthy mice. As previously shown in human monocytes [12], this protocol is a strong promoter of pro-lymphatic differentiation as indicated by analysis on day 6 of differentiation that shows >85% of cells expressing LEC markers collectin-12 (Colec12), integrin-alpha9 (Itga9), Lyve-1, and Pdpn as compared with <10% in freshly-isolated *ex-vivo* cells (P-values <0.001; Fig 1B and 1C). Compared with *ex-vivo* cells, the mean fluorescent intensity (MFI) was elevated for differentiated cells with statistically significant differences for all examined LEC markers except Colec12 (Fig 1D). Upregulation of LEC proteins coincided with increased pan-vascular markers Vegfr-1 and CD105 expressed by both lymphatic and blood vascular endothelial cell (BEC) progenitors (Fig 1E and 1F). In contrast, the proportion of cells expressing BEC-specific markers podocalyxin (Podxl), CD31, neuropilin-1 (Nrp-1), CD146, Meca-32, and Vegfr-2 remained below the 20% (Fig 1E, right panel) and MFI for nearly all BEC-specific markers was unchanged or decreased in differentiated cells compared with *ex-vivo* (Fig 1F and 1G). These data suggest that the CSF-1/LPS protocol induces myeloid-endothelial progenitors with predominant lymphatic phenotype.

### Pro-lymphatic differentiation coincides with IL-10-dependent shift to M2-biased monocytic progenitors

We previously showed that M-LECP recruited to clinical BC are part of M2-type TAM population [16] known to be induced by immunosuppressive Th2 cytokines IL-4, IL-13 or IL-10 [45–47]. We also showed that IL-10 and its receptor IL-10R are co-regulated by CSF-1/LPS treatment, which created an autocrine loop and promoted expression of lymphatic markers [25]. This led to the hypothesis that promotion of pro-lymphatic differentiation and acquisition of the M2 phenotype are both regulated by IL-10 pathway activated in CSF-1/LPS treated myeloid precursors.

**Fig 2:**
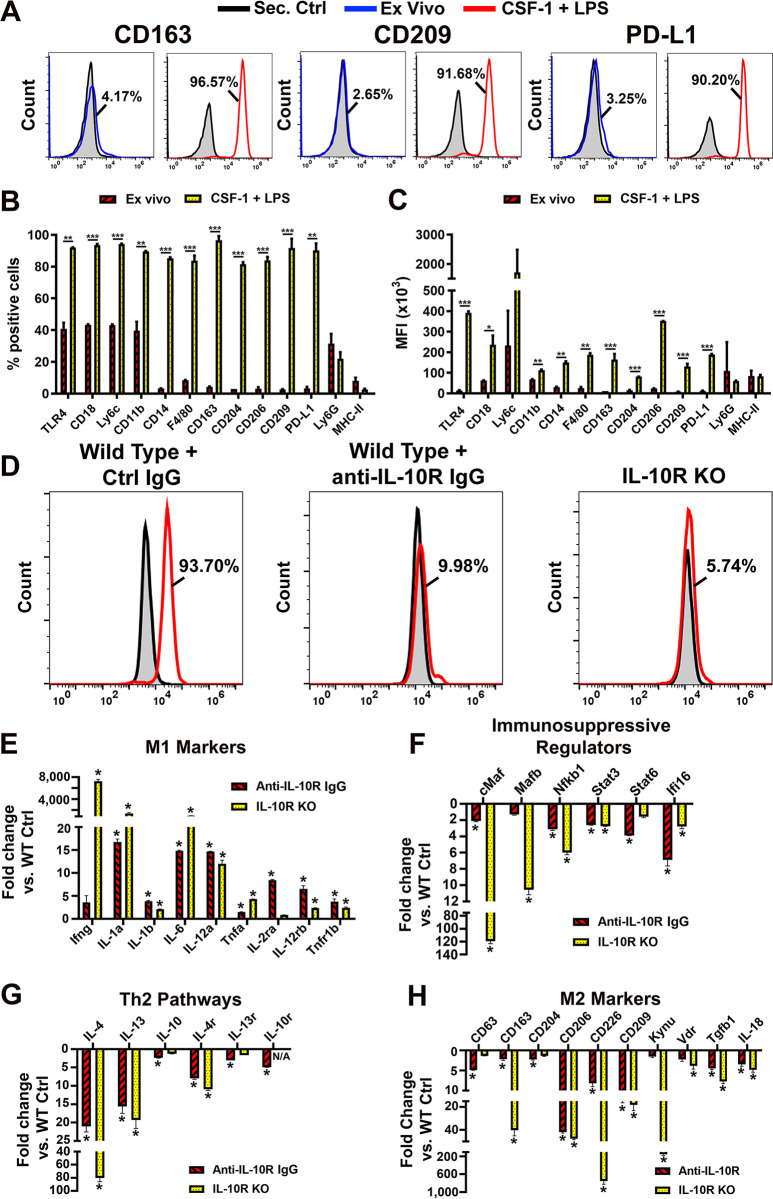
Pro-lymphatic differentiation coincides with IL-10 dependent shift to M2 phenotype. (A) Representative histograms demonstrating expression of M2-specific myeloid markers CD163, CD209, and PD-L1 in freshly-isolated BM cells (Ex-vivo, blue line) and differentiated cells (CSF-1 + LPS, red line). Numbers in black font indicate the percentage of cells expressing an M2-specific marker. (B) The mean percentage of positive cells and (C) MFI (x10^3^) of M2 marker expression in *ex-vivo* and differentiated cells. (D) Histograms demonstrating the expression of CD204 in CSF-1/LPS differentiated cells derived from wild-type C57BL/6 mice in the presence of rat isotype-matched control (left panel) or anti-IL-10R antibody (center panel). The right panel shows CD204 expression in identically differentiated cells from IL-10R knockout (KO) mice. Secondary controls are shown by the grey area outlined by black line. Transcriptional analysis determined by qPCR on day 6 of differentiation of anti-IL-10R antibody-treated BM cells compared with rat control antibody, and BM cells from IL-10R-/- compared with those from WT mice. Analyzed markers included those for (E) M1 phenotype, (F) immunosuppressive regulators, (G) Th2 regulators, and (H) M2 phenotype. Expression in WT cells in the absence or presence of control rat IgG was taken as 1. Stars represent statistical significance determined by Student’s t-test with P-values indicated by *<0.05, **<0.01, and ***<0.001. All experiments were performed in duplicate and reproduced at least twice.

To test this hypothesis, we compared CSF-1/LPS induction of LEC and M2 markers in control BM cells and those with blocked IL-10 pathway or lacking IL-10R. Differentiation by CSF-1/LPS caused strong upregulation of CD163, CD204, CD206, CD209, and PD-L1 in 84%-96% of BM cells as compared with 5–10% positive cells in naïve population (Fig 2A–2C). Pan-macrophage identifiers such as TLR4, CD18, and CD11b also increased from ~40% in naïve cells to 90–95% in differentiated cells (Fig 2B). In contrast, the MFI and the percentage of positive cells for MHC class II antigen associated with stimulatory M1 phenotype were decreased upon differentiation. Bias to monocytic *versus* granulocytic development was indicated by high expression of Ly6C in up to 95% of the cells as opposed to a minor Ly6G^+^ fraction (Fig 2B). These changes were IL-10 dependent as exemplified by 89–94% decrease in expression of an M2 marker CD204 in cells treated with anti-IL-10R antibody or derived from IL-10R knockout (KO) mice (Fig 2D). The flow cytometry data were consistent with transcriptional shifts detected by qPCR (Fig 2E–2H). The latter analyses showed that blocking or deleting IL-10R during CSF-1/LPS induced differentiation significantly increased M1 and downregulated M2 markers (Fig 2E–2H). Mediators of the M2 bias such as transcription factors c-Maf [48], MafB [48], NFKB1 [49], Stat3 [50], and Stat6 [51] were all downregulated in the absence of functional IL-10 pathway compared with control cells (Fig 2F). This corresponded to substantial 60–90% declines in all Th2 regulators (Fig 2G) ultimately leading to suppression of the M2 phenotype. Collectively, these data show that CSF-1/LPS differentiation of myeloid-lymphatic progenitors is closely associated with acquisition of M2 immunosuppressive phenotype, and that both lymphatic [25] and myeloid (Fig 2) developments are regulated by the IL-10 pathway.

### Myeloid-lymphatic progenitors induced by CSF-1/TLR4 activation retain and increase expression of stem/progenitor cell markers

**Fig 3:**
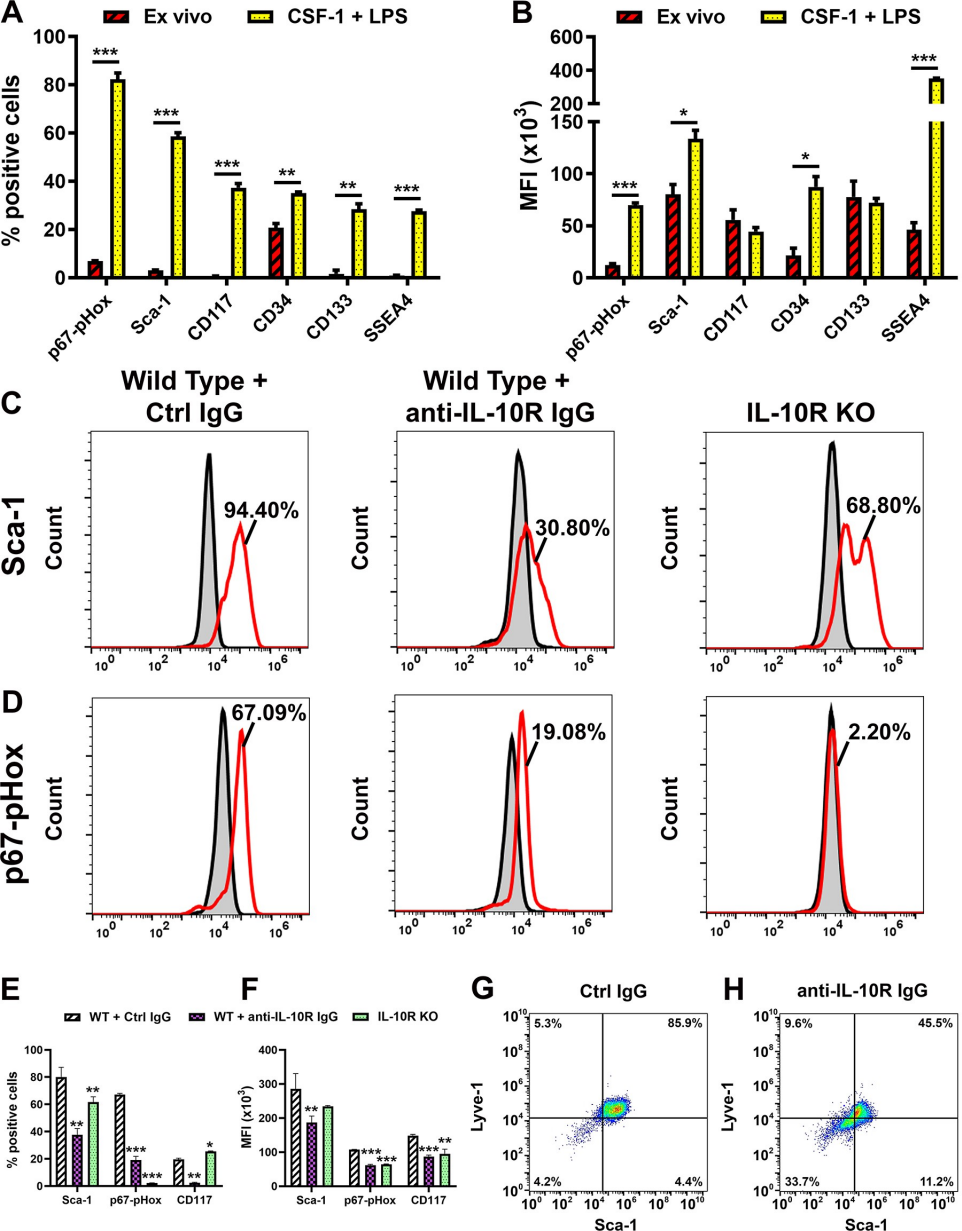
Acquisition of M2 phenotype coincides with IL-10 dependent upregulation of stem markers. BM cells were differentiated using standard protocol followed by analysis of stem cell markers p67-pHox, Sca-1, CD117, CD34, CD133, and SSEA4. (A) The mean percentage of positive cells and (B) MFI (x10^3^) were compared in freshly-isolated and differentiated cells using flow cytometry. Representative histograms demonstrating expression of (C) Sca-1 and (D) p67-pHox in WT differentiated BM cells in the presence of control rat IgG, anti-IL-10R IgG, and differentiated cells from IL-10R KO mice. The numbers indicate the percentage of marker-positive cell population shown by red line. Secondary controls are shown by the grey area outlined by black line. (E) The mean percentage of positive cells and (F) MFI (x10^3^) of marker expression in each panel. (G, H) Co-expression of a lymphatic marker Lyve-1 and a stem cell marker Sca-1 in differentiated cells from WT in the presence of control and anti-IL-10R antibody. Stars represent statistical significance determined by Student’s t-test with P-values indicated by *<0.05, **<0.001, and ***<0.001. All experiments were performed in duplicate and reproduced at least twice.

A widely held view is that maturation of precursors towards specific lineages is associated with downregulation of stem/progenitor cell markers [52, 53]. We tested this concept by analyzing expression of specific markers for stem and early progenitor cells in CSF-1/LPS treated mouse BM cells. In contrast to an anticipated decrease of stem markers, we detected higher expression of all six examined targets in differentiated compared with *ex-vivo* cells. This is indicated by changes in both the percentage of positive cells and MFI (P-values <0.01 to 0.001; Fig 3). Cells with expression of markers signifying self-renewal and undifferentiated status such as p67-pHox [54], CD34 [55], SSEA4 [56], CD133 [57], c-Kit (CD117) [58], and Sca-1 [59] comprised 30 to 80% of the differentiated population compared with only 2%-4% prior to CSF-1/LPS treatment (Fig 3A). The highest upregulated marker Sca-1 was detected in 94.4% of differentiated cells. The number of positive cells for Sca-1 and other stem markers significantly decreased in the presence of anti-IL-10R antibody and in BM cells from IL-10R-/- mice (Fig 3C–3F). Moreover, the number of double-positive Sca-1^+^/Lyve-1^+^ cells dropped by half from 85.9% to 45.5% in WT cells in the presence of anti-IL-10R antibody compared with control IgG (Fig 3G and 3H). Collectively, these data indicate that both pro-lymphatic and myeloid developments induced by IL-10 are closely associated with increase in stem cell properties suggesting transition to multipotency rather than commitment to a specific lineage.

### IL-10 pathway promotes multipotency in CSF-1/LPS activated BM cells

**Fig 4:**
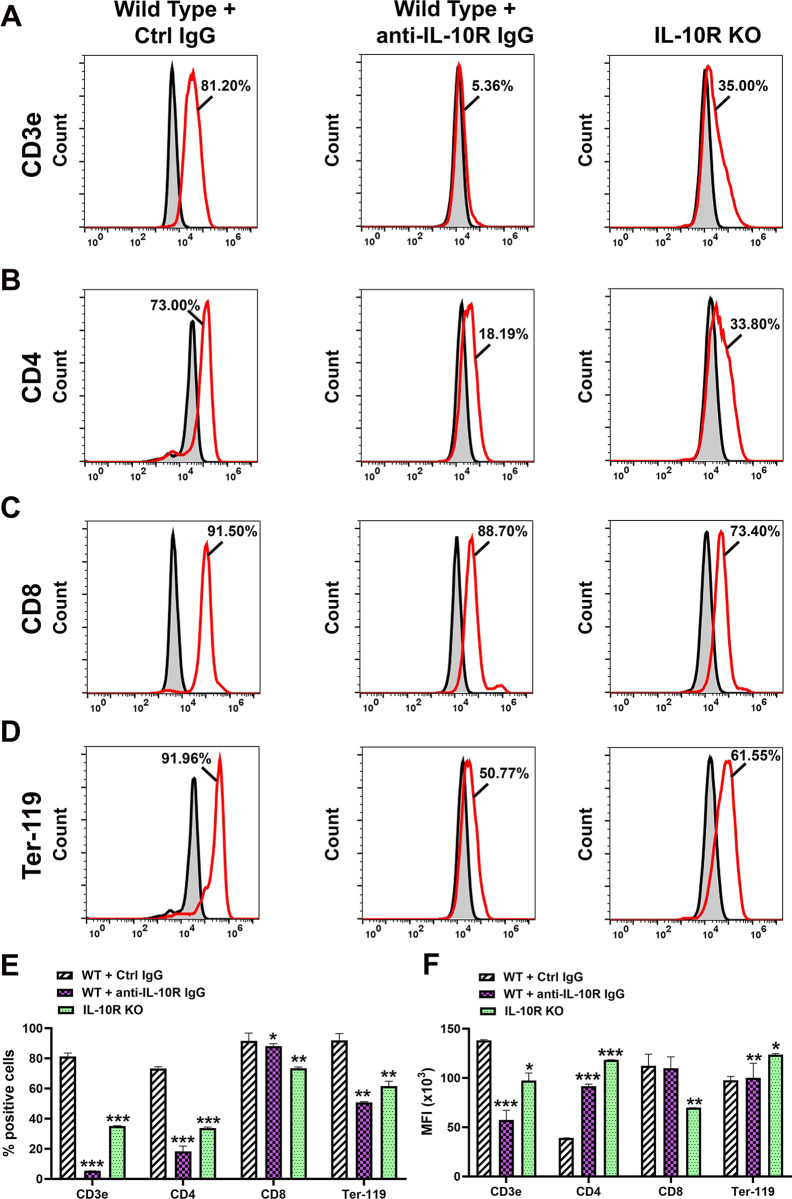
CSF-1/LPS upregulates markers of multiple lineages in an IL-10 dependent manner. Representative histograms demonstrating expression of (A) CD3e, (B) CD4, (C) CD8, and (D) Ter-119 in BM cells from WT mice in the presence of control (left panel) and anti-IL-10R antibody (center panel) as well as in BM cells from IL-10R-/- mice (right panel). All cells were differentiated using standard CSF-1/LPS protocol as described under Methods. Numbers indicate the mean percentage of marker-positive cells in each condition. Secondary controls are shown by the grey area outlined by black line. (E) The mean percentage of positive cells and (F) MFI (x10^3^) of indicated markers in differentiated WT cells treated with control or anti-IL-10R antibody and in identically differentiated IL-10R-/- BM cells. Statistical significance of differences in marker expression was determined by Student’s t-test with P-values indicated by *<0.05, **<0.001, and ***<0.001. All experiments were performed in duplicate and reproduced at least twice.

To test the hypothesis that IL-10 promotes multipotency, we compared expression of cell lineage markers other than lymphatic or myeloid in *ex-vivo* and CSF-1/LPS differentiated BM cells from WT cultured with control rat IgG; WT cultured with anti-IL-10R IgG; and BM cells from IL-10R-/- mice. We found that markers of T-lymphocytes (CD3, CD4, and CD8) as well as Ter-119, a marker of erythroid cells, were increased up to 92% in differentiated WT cells cultured with control IgG (Fig 4A–4E) compared with 5–11% in an *ex-vivo* population (S1 Fig). The MFI of CD4 and CD8 was also significantly elevated (Fig 4F) along with myeloid (Fig 2) and lymphatic-specific proteins (Fig 1). Apart from CD8, upregulation of the T-cell and erythroid lineage markers was IL-10 dependent as evident by a substantial decrease in the presence of anti-IL-10R antibody and in BM cells lacking IL-10R (Fig 4). Blocking the IL-10 pathway caused a 20% decrease in CD8^+^ cells in BM cells from IL-10R-/- mice whereas CD3e, CD4, and Ter-119 were suppressed by 93%, 75%, and 45% by anti-IL-10R antibody, respectively, compared with corresponding controls. These data suggest that the CSF-1/TLR4/IL-10 axis generates intermediate precursors with the potential to produce progenitors for lymphatic endothelial and some hematopoietic lineages.

### Activation of IL-10 in M2 myeloid precursors promotes the developmental potential for several hematopoietic and endothelial lineages

**Fig 5:**
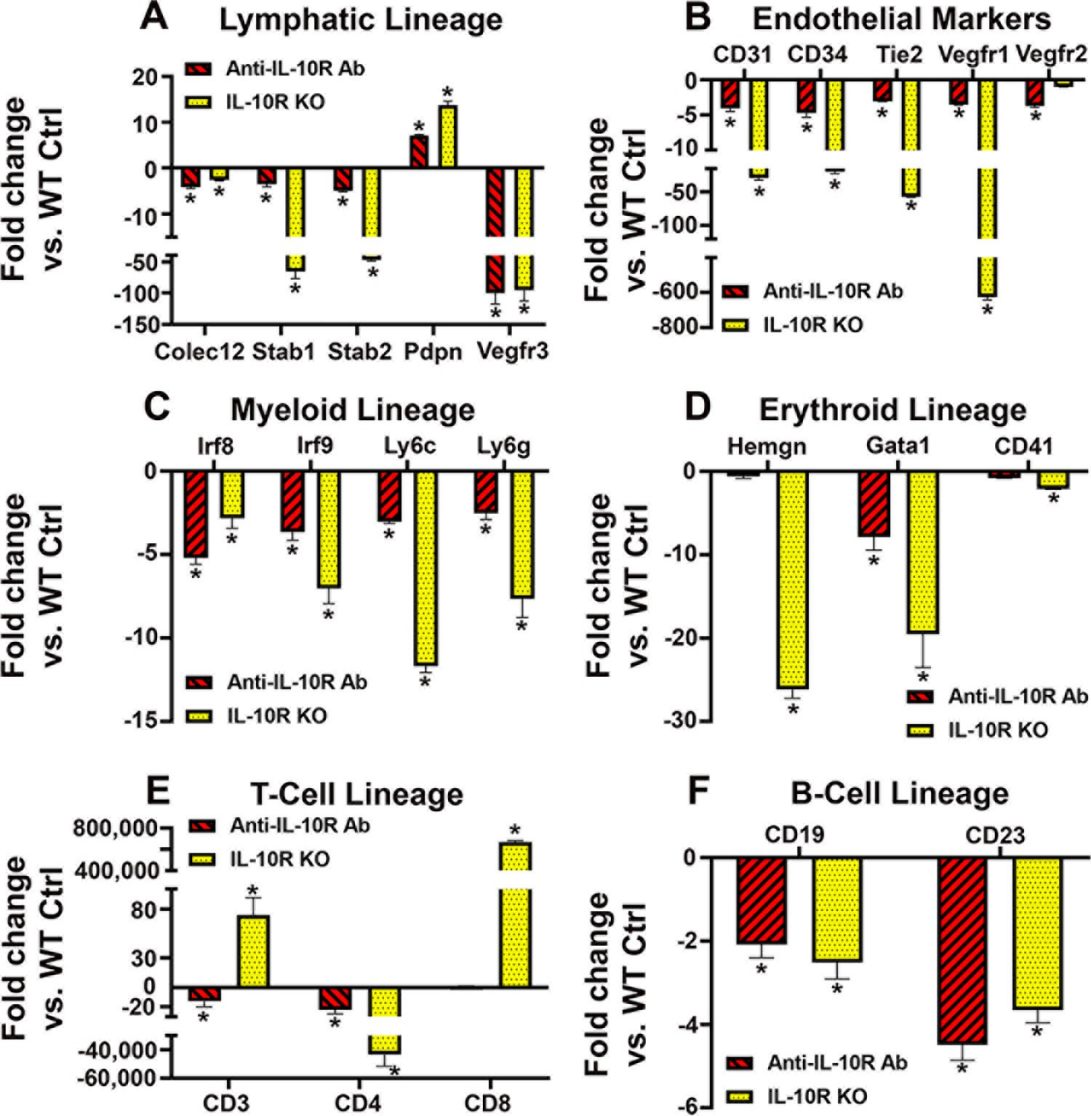
IL-10 pathway regulates transcription of specific markers of multiple lineages in BM myeloid precursors. Marker expression levels in CSF-1/LPS differentiated BM cells from WT mice in the presence of anti-IL-10R antibodies and from IL-10R KO mice were compared with the levels in WT cells treated with a control antibody. Cells harvested on the sixth day of differentiation were analyzed by RT-qPCR for mRNA expression of markers of (A) lymphatic-specific, (B) endothelial, (C) myeloid, (D) erythroid, (E) T-cell, and (F) B-cell lineages. All analyses were performed in triplicate for each target, normalized to β-actin, and independently reproduced twice. Data for each target are reported as the mean fold-change in treated cells compared with the WT cells treated with control rat IgG ± S.D. Statistical significance of expression changes compared with control group was determined by Student’s t-test with P-values indicated by *<0.05, **<0.01, and ***<0.001.

To examine a transcriptional role of the IL-10 pathway in multipotency, we used qPCR to determine *de novo* expression of specific markers of various lineages in the presence of a blocking anti-IL-10R antibody and in BM cells lacking IL-10R. The β-actin normalized Ct values from cells with blocked or deleted IL-10R were compared with WT cells in the presence of control rat IgG (control IgG) and WT cells with no additions (control for IL-10R-/-), respectively. We found transcriptional analyses to be highly consistent with flow cytometry data with both demonstrating a direct role of IL-10 in induction of markers of multiple lineages. As shown in Fig 5, transcripts for lymphatic, endothelial, myeloid, erythroid, T-cell, and B-cell lineage-specific genes were significantly reduced in both anti-IL-10R antibody treated cells and those lacking this receptor, compared with respective controls. Among LEC markers, Pdpn transcript was the only target insensitive to IL-10 inhibition which is consistent with previous observations that Pdpn expression is independent of TLR4 [25]. The other outlier was CD8, a marker of cytotoxic T-cells, which is consistent with an increase in immuno-stimulatory responses upon suppression of Th2 pathways [60]. However, T-cell lineage markers CD3e and CD4 were decreased upon inhibition of IL-10R (Fig 5E). Both IL-10 deficient models were in complete agreement with regard to suppression of multi-lineage differentiation although permanent deletion of IL-10R in BM from KO mice had stronger effects compared with that of anti-IL-10R antibody. These data strongly support our contention that the primary role of CSF-1/TLR4 activated IL-10 pathway in early myeloid precursors is to confer developmental potential for multiple lineages including production of myeloid-lymphatic progenitors.

### Lymphatic progenitors induced by tumors in BM *in vivo* express markers of multiple hematopoietic lineages

**Fig 6:**
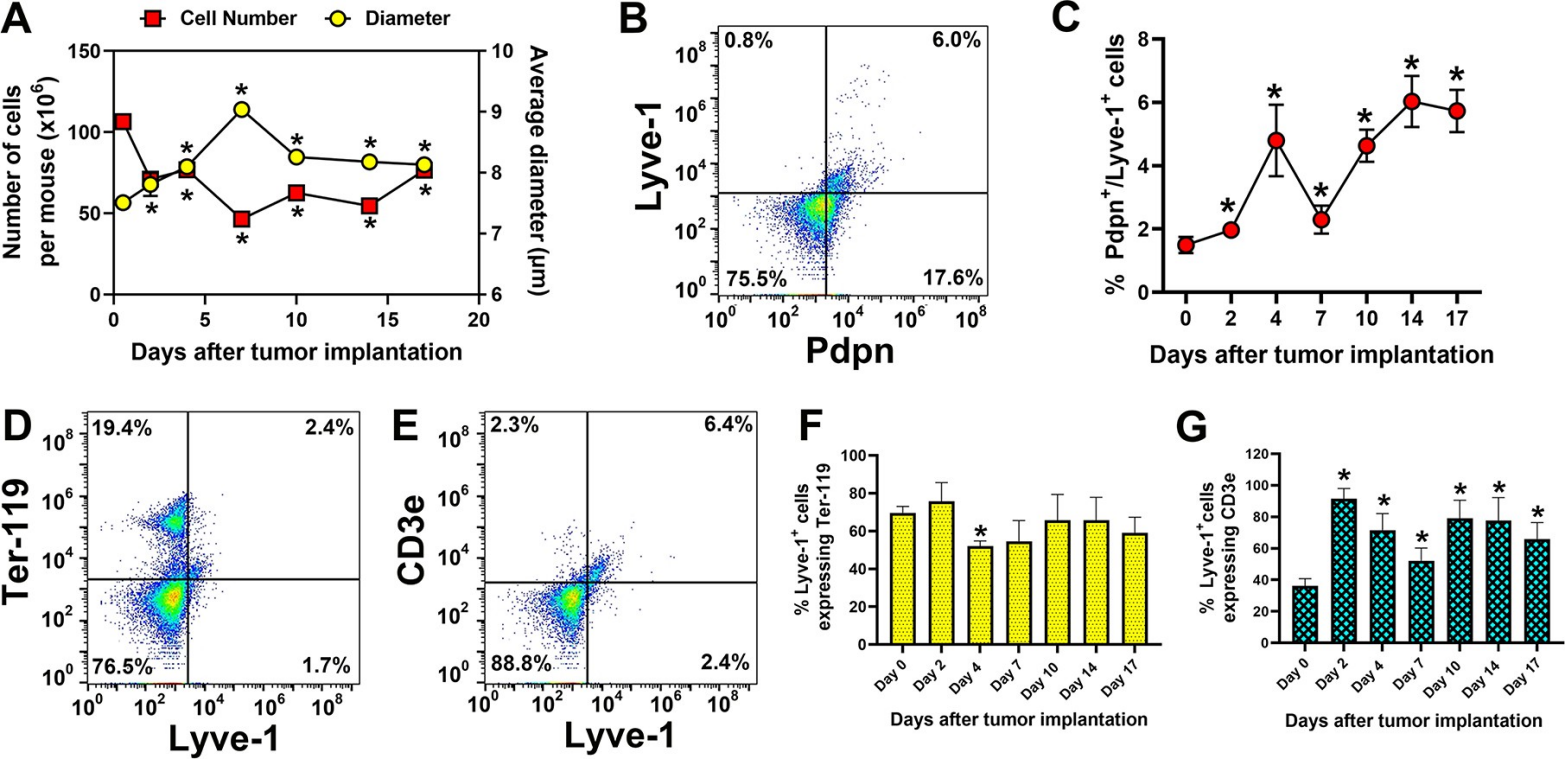
Mouse syngeneic breast tumors *in vivo* induce progenitors with multi-lineage markers. BALB/c mice were orthotopically implanted with EMT6 breast cancer cells (N = 2 mice per timepoint) with one group serving as a tumor-free control. BM was extracted from the control group on day 0 and from experimental groups on days 2, 4, 7, 10, 14, and 17 after tumor implantations. After determining cell number and their diameter, cells were double-stained for Lyve-1, Pdpn, Ter-119, and CD3e in different combinations and analyzed using flow cytometry. BM from all mice per timepoint were pooled and evaluated in triplicate. (A) The mean values for BM cell numbers and cell diameter on different days post tumor implantation. (B) A representative flow cytometry dot plot of double-positive Pdpn^+^/Lyve-1^+^ cells in BM harvested on the 14^th^ day post tumor implantation. (C) The mean percentage of BM cells of Pdpn^+^/Lyve-1^+^ double-positive cells in the absence of tumor (day 0) and on different days after tumor implantation. A representative dot plot of double-positive (D) Ter-119^+^/Lyve-1^+^ and (E) CD3e^+^/Lyve-1^+^ cells in BM harvested on the 7^th^ day post tumor implantation. The mean percentage of (F) Lyve-1^+^/Ter-119^+^ or (G) Lyve-1^+^/CD3e^+^ cells from total Lyve-1^+^ cell in the BM was determined in tumor-free and EMT6 bearing mice on different days post implantation. All values are presented as the mean of triplicate ± S.D. Statistical significance of differences between control (no tumor) and tumor-induced changes was determined by Student’s t-test with * indicating P-values <0.05.

To test the relevance of data obtained from the *in vitro* model to *in vivo* M-LECP differentiation, we analyzed BM cell surface profiles in mice implanted with syngeneic EMT6 breast tumors. BM cells were collected from tumor-free mice (day 0 control) and on the days 2, 4, 7, 10, 14, and 17 post tumor implantations. This was followed by analyses for total cell number per two femurs; cell diameter; and expression of lymphatic (Lyve-1, Pdpn), erythroid (Ter-119) and pan T-cell lymphoid (CD3e) proteins. We found that tumors induced drastic changes in numbers and diameters of BM cells. This was particularly noticeable during the first week post-implantation when the total cell number dropped by half, presumably due to rapid mobilization of progenitors to the blood (Fig 6A). The losses reached >60% by day 7 followed by gradual stabilization and a modest increase in the next two weeks. The drastic cell exit coincided with increased cell diameter by 25% that typically signifies differentiation. However, in all days of analyses the rate of production of new progenitors failed to balance the rate of their release, probably due to exponential demands of a growing tumor. Due to disbalance between the production and the release rates of BM cells *in vivo*, the Lyve-1^+^/Pdpn^+^ fraction of lymphatic progenitors accounted only for 6% of total cells (Fig 6B), which is significantly lower than in an *in vitro* assay shielded from tumor-induced mobilization. However, the multipotent profile of myeloid-lymphatic progenitors established in *in vitro* studies (Figs 1–5) has been recapitulated in this *in vivo* setting. For instance, 70–80% of Lyve-1 positive cells co-expressed Ter-119 and CD3e for the duration of the experiment and the percentage of multi-positive cells significantly exceeded the baseline (Fig 6C–6G). These findings confirm that multipotent status of BM progenitors identified in the *in vitro* model (Figs 4 and 5) is faithfully reproduced in the BM of tumor-bearing mice.

### Tumor-recruited lymphatic progenitors retain the myeloid phenotype but downregulate markers of other hematopoietic lineages in human breast cancer ZR-75 model

To date, M-LECP in mouse and human tumors have been analyzed only for myeloid-specific and lymphatic markers but not for indicators of other lineages. Since our data from *in vitro* and *in vivo* analyses suggested multipotency of M-LECP in the BM, we wished to determine whether tumor-recruited progenitors continue to maintain this status in tumors. To address this question, we used two orthotopic models of breast cancer: human ZR-75 xenografts grown in SCID mice and mouse syngeneic EMT6 cell line implanted into BALB/c mice. We also used two methods to identify tumor-recruited M-LECP based on either GFP expression in *in vitro* differentiated progenitors derived from BM of GFP^+^ mice or co-expression of Lyve-1 and CD11b markers in the absence of GFP. Adoptively transferred GFP^+^ M-LECP were defined as exogenous progenitors whereas GFP-negative cells with myeloid-lymphatic phenotype were identified as endogenous progenitors. This analysis allowed us to confirm the BM origin of tumor-infiltrating M-LECP and compare the tumor behavior of progenitors produced by *in vitro* differentiation *versus* natural induction by tumors *in vivo*.

**Fig 7:**
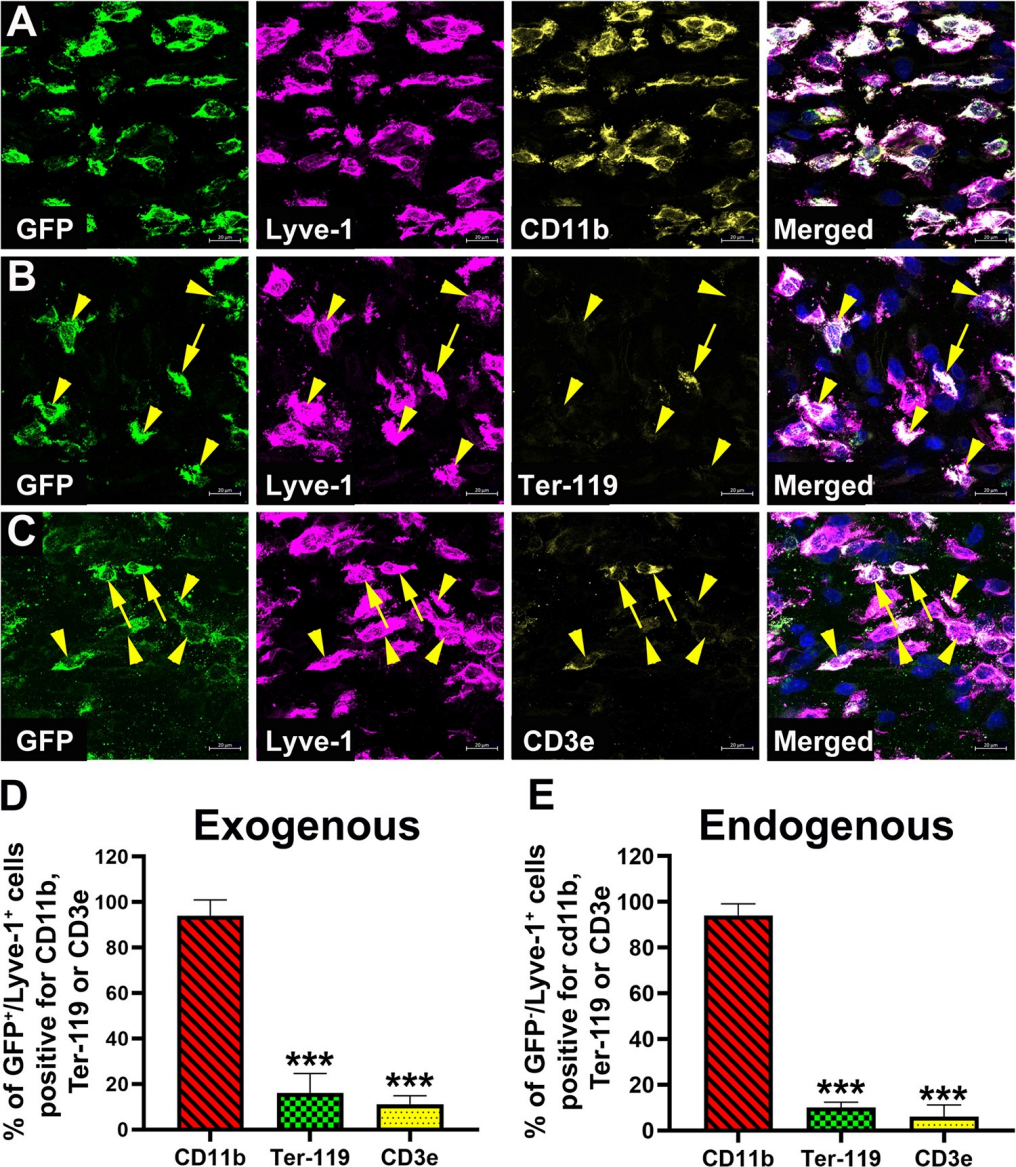
M-LECP recruited to ZR-75 human tumors retain myeloid-lymphatic identity but downregulate T-cell and erythroid markers. Human ZR-75 breast tumor cells were orthotopically implanted into SCID mice (N = 5). When tumors reached ~300mm^3^, mice were infused i.v. with 1.5x10^6^ of GFP^+^ M-LECP differentiated *in vitro* using standard CSF-1/LPS protocol. Four weeks after transfer, tumors were excised and triple-stained for GFP, Lyve-1, and (A) CD11b, (B) Ter-119 or (C) CD3e. Nuclei (blue) shown in merged images were visualized by Hoechst stain. Yellow arrowheads indicate GFP^+^/Lyve-1^+^ cells that lack markers of other lineages. Cells with rare co-expression of erythroid or lymphoid markers in GFP^+^/Lyve-1^+^ cells are indicted by yellow arrows. All images were acquired at 400X magnification. (D) The mean percentage of GFP^+^/Lyve-1^+^ cells expressing CD11b, Ter-119 or CD3e was determined on 3–4 tumor sections each derived from individual mice. A minimum of 100 GFP^+^/Lyve-1^+^ cells identified in multiple sections were analyzed to calculate the percentage cells positive for each marker. (E) Identical analysis was applied to native Lyve-1^+^ cells lacking GFP expression. Results are presented as the means ± S.D. “Exogenous” and “Endogenous” labels refer to adoptively transferred (GFP^+^) or tumor-induced host M-LECP (GFP^-^), respectively. Statistically significant differences between Lyve-1^+^ cells with myeloid and other markers were determined by Student’s t-test with P-values of <0.001 indicated by triple stars.

We found that both exogenous and endogenous M-LECP in both models of breast cancer display an identical pattern of retaining the myeloid marker CD11b but downregulating markers of other lineages (Fig 7). GFP^+^/Lyve-1^+^/CD11b^+^ macrophages were identified on tumor sections by triple staining and evaluated for co-expression of erythroid and T-cell lymphoid markers. As shown in Fig 7, more than 90% of tumor-recruited GFP^+^ cells co-expressed myeloid CD11b and lymphatic Lyve-1 markers. In contrast, less than 10% of GFP^+^ cells expressed an erythroid Ter-119 or a pan T-lymphocyte CD3e markers (Fig 7D). Analysis of GFP^+^ BM cells prior to injection determined that myeloid, erythroid, T-cell, and lymphatic markers were highly expressed (up to 90%) *in vitro* (Fig 4B and S2 Fig) suggesting that low or absent expression of CD3e and Ter-119 in tumor-residing M-LECP was due to *in vivo* induced suppression. Since GFP is a trackable marker, tumor-residing GFP^+^/CD11b^+^/Lyve-1^+^ cells that lacked T-cell and erythroid markers were clearly derived from an initially positive subset generated *in vitro* rather than from unknown precursors. Endogenous GFP-negative CD11b^+^/Lyve-1^+^ progenitors showed the same pattern, that is, largely lacked CD3e and Ter-119 (Fig 7E).

### Retention of myeloid-lymphatic phenotype along with reduction of erythroid and T-cell markers is replicated in syngeneic EMT6 breast cancer model

**Fig 8:**
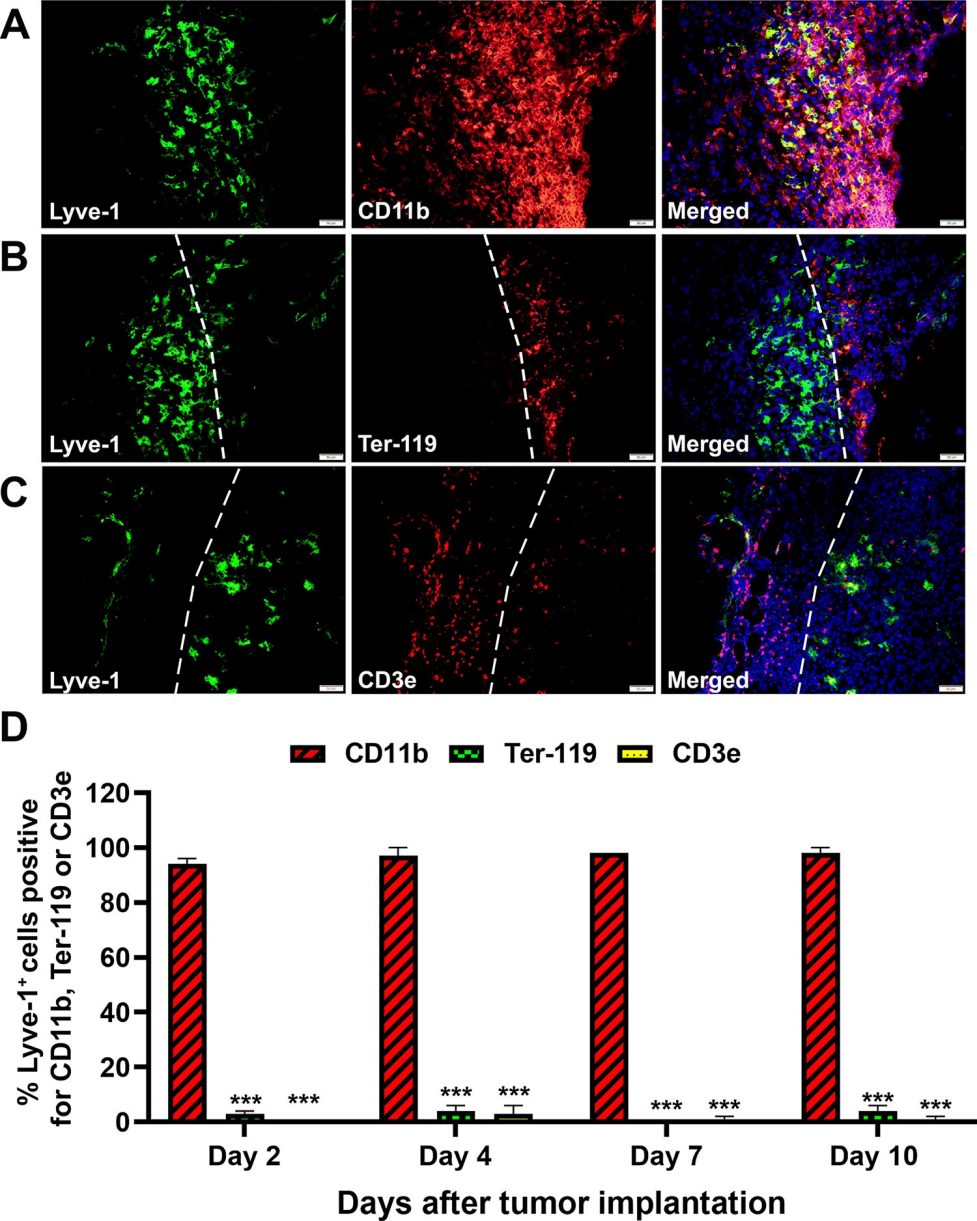
M-LECP recruited to mouse EMT6 tumors are distinct from co-mobilized Ter-119^+^ or CD3e^+^ progenitors. BALB/c mice were orthotopically implanted with EMT6 breast cancer cells in groups of 4 mice per timepoint. Tumors were excised on days 2, 4, 7 and 10 post-implantations, sectioned and stained for Lyve-1 in combination with (A) CD11b, (B) Ter-119, or (C) CD3e. Nuclei (blue) in merged images were visualized by Hoechst stain. All images were acquired at 400X magnification. (D) The mean percentage of Lyve-1^+^ cells co-expressing CD11b, Ter-119 or CD3e was determined on tumor sections from individual mice (N = 4) with a minimum of 100 Lyve-1^+^ cells analyzed for each marker. Results are presented as the means ± S.D. Statistically significant differences between Lyve-1^+^ cells with myeloid and other markers were determined by Student’s t-test with P-values of <0.001 indicated by triple stars.

The immunocompetent, syngeneic EMT6 tumor model replicated the progenitor behavior in a xenograft model: nearly 100% of EMT6-recruited Lyve-1^+^ cells expressed CD11b but only 1–3% co-expressed either Ter-119 or CD3e (Fig 8). This pattern was observed regardless of the day of post-implantation suggesting either rapid intratumoral switch to a specific lineage or maturation prior to tumor entry. Physical segregation of progenitors into distinct lineage-specialized subsets was indicated by clusters of Ter-119 and CD3e positive cells with red color that were clearly separated from green-labeled Lyve-1^+^ cells (dotted white line, Fig 8B and 8C). As in the ZR-75 model, CD11b^+^/Lyve-1^+^ cells were more abundant than either CD3e^+^ or Ter-119^+^ cells. These data suggest that tumors can determine a composition of recruited progenitors that achieve advanced maturation either prior to tumor arrival or upon direct influence of TME.

## Discussion

Myeloid-lymphatic endothelial cell progenitors (M-LECP) are BM-derived immunosuppressive M2-type cells that co-express stem and lymphatic markers, are capable of increasing tumor LVD in BC models [61], and are associated with increased LN metastasis in BC patients [16]. We previously showed that M-LECP can be generated from BM cells *in vitro* by activation of CSF-1 and TLR4 pathways [12]. This protocol activates the IL-10 pathway that induces the M2 immunosuppressive and lymphatic phenotypes [25]. In addition to well-established M2 polarization of TAMs [62] and BM myeloid cells [63], IL-10 also supports differentiation of a wide range of lineages generating epithelial, endothelial, hematopoietic, and neuronal cell types [26, 27]. Given the broad involvement of IL-10 in generation of multiple lineages, we sought better understanding of its specific role in induction of lymphatic progenitors. To this end, we compared expression of markers identifying progenitors for various hematopoietic lineages in CSF-1/LPS differentiated cells *in vitro* as well as in the BM and tumors *in vivo*. The main conclusions of this study are: (1) IL-10 expressed in the BM induces multipotent progenitors as suggested by co-expression of specific markers for at least four lineages: myeloid-macrophage, lymphatic endothelial, T-cell lymphoid, and erythroid; (2) IL-10 dependent induction of multipotency coincides with upregulation of stem cell markers (i.e., plasticity) and emergence of immunosuppressive M2 phenotype; (3) BM in tumor-bearing mice replicates generation of multipotent CD11b^+^/Lyve-1^+^/Ter-119^+^/CD3e^+^ cells *in vitro*; and (4) compared with BM, tumor-residing progenitors display advanced maturity as evidenced by physically separated lineage-specific subsets residing at distinct tumor locales.

Our data suggest that BM-derived IL-10 induces multipotent progenitors. This is in line with prior studies that implicated IL-10 in differentiation of a variety of hematopoietic and non-hematopoietic lineages [28, 40, 41, 64] including multipotent MSC [35, 38]. It has also been shown to promote differentiation of blood vascular [29] and lymphatic endothelial lineages [25] that can derive from both HSC-myeloid precursors [12] and MSC [65]. Our data suggest that IL-10 can also promote the erythroid lineage (Figs 4 and 5). Such broad involvement in cell differentiation suggests a role in generating multipotent precursors rather than a specific lineage. This conclusion is supported by several lines of evidence and arguments. Conceptually, BM released cells are expected to maintain plasticity to allow the local environment to finalize the properties of progenitors needed for repair of a specific site. In line with this concept, IL-10 has been shown to promote proliferation, survival and self-renewal of multipotent HSC [26] and MSC [38] that are located at the top of hierarchical trees and can give rise to multiple cell types. Mechanistically, IL-10 is well-equipped to create the lineage diversity as it can control access to DNA through histone-3 acetylase H3Ac [66], histone-3 methylase H3K4me4 [67], CCAAAT-enhancer binding protein (C-EBP) [68], and other epigenetic regulators [69]. H3K4me4 significantly contributes to stem cell potency [70] whereas C-EBP a key player in differentiation of early myeloid precursors with the capacity to produce multiple lineages [71]. Importantly, IL-10 dependent epigenetic regulators are regulated by LPS-activated TLR4 pathway [67, 72], an important regulator of BM-produced M-LECP [12]. Convergence of cell plasticity with acquisition of the lymphatic phenotype is evident in our study that demonstrates concurrent elevation of LEC-specific and stem cell markers such as Sca-1, CD133, and c-Kit (Fig 3). Expression of these stemness and self-renewal markers in mouse [65, 73] and human [14, 74, 75] lymphatic progenitors suggests their plasticity after release from the BM. Taken together, this evidence supports our hypothesis that IL-10 generated multipotent progenitors in the BM can harbor the potential to progress to various lineages outside of the original production site.

We also show here that IL-10 dependent pro-lymphatic reprogramming coincides with acquisition of immunosuppressive M2 phenotype (Fig 2). This is consistent with the observed M2 status of lymphatic progenitors in clinical BC [16] and other human tumors [13]. The well-known properties of M2 macrophages such as efficient mobility, invasiveness, and matrix degradation can significantly help M-LECP to access tumor lymphatic vessels. Combining pro-migratory behavior of M2-TAMs with multipotency and immunosuppression might further increase functionality of M-LECP and other BM progenitors. Multipotency confers flexibility of recruited progenitors to support specific tissue needs while suppression of cell-destructive immune responses is essential for tissue repair. It therefore makes a physiological sense to equip regenerative BM cells with strong migratory/invasive capacities along with the mechanisms to suppress stimulatory immune responses and maintain broad developmental potential.

The BM responses to tumors have received much less attention than analysis of TME. Here, we show that implantation of 1.5x10^6^ of breast tumor cells depletes nearly half of the entire BM cell inventory in just two days (Fig 6A). While the total number of BM cells in tumor-bearing mice remained significantly lower than the baseline for the duration of the experiment, the fraction of lymphatic progenitors steadily increased reaching 300% of the level prior to tumor implantation (Fig 6C). In line with *in vitro* findings (Fig 4), ~80% of Lyve-1^+^ cells in BM co-expressed Ter-119 and CD3e (Fig 6F and 6G). This evidence supports our hypothesis that BM produces early multipotent progenitors that subsequently mature at extramedullary or final destination sites.

Our study demonstrated significant differences in marker profiles of Lyve-1^+^ cells in BM and tumors: BM-residing Lyve-1^+^ cells co-expressed multipotent markers whereas in tumors nearly all Lyve-1^+^ cells lacked Ter-119 and CD3e (Figs 7 and 8). While we cannot be certain that tumor-residing Lyve-1^+^ cells are derived directly from the BM generated Lyve-1^+^ cells, our adoptive transfer experiment is consistent with this interpretation. In this experiment, 86% to 94% of differentiated GFP^+^ cells were positive for Lyve-1, Ter-119 and CD3e immediately *prior* to transfer (Fig 4 and S2 Fig), but only 10% of tumor-residing GFP^+^ cells displayed this profile 4 weeks after transfer (Fig 7). Analysis of this GFP^+^ trackable cell population with nearly 90% overlapping expression for markers of three different lineages clearly demonstrates the uniformity of co-expression prior to transfer. However, tracking tumor-recruited GFP-positive cells showed that Lyve-1^+^, Ter-119^+^ and CD3e^+^ subsets were segregated and localized to distinct intratumoral sites (Fig 8). This evidence suggests that BM-generated myeloid progenitors can undergo further specialization either at an extramedullary organ (e.g., spleen) or upon tumor arrival. M-LECP derivation from originally multipotent precursors is supported by studies showing generation of arterial and venous endothelial progenitors from multipotent monocytes [76, 77] that were also able to give rise to neuronal [78] and mesenchymal [79] cells. Lymphatic progenitors can also derive from MSC [80] suggesting that either hematopoietic or mesenchymal multipotent cells contribute to expansion of tumor lymphatic vasculature. Taken together with our findings, these studies suggest that BM progenitors might be initially multipotent while the advanced or terminal determination is achieved at an intermediate storage or final destination site. Such flexibility afforded in the BM presumably by IL-10 can later support optimal adaptation of endothelial progenitors to the specific needs of tumor vascular bed.

One of the unresolved questions of this study is when and where multipotent progenitors transit to more committed cells. We detected distinct progenitor populations as early as two days after tumor implantation (Fig 8). This short time suggests that differentiation of BM-released monocytic progenitors might occur in spleen or another organ prior to tumor arrival. The concept of the spleen as an intermediate storage and differentiation site is supported by the studies that showed mobilization of BM-produced early progenitors to this organ [81–83]. This event was followed by acquisition of new or enhanced properties such as expression of chemotactic receptors [84] and improved survival [85]. That said, tumors might also influence the composition of residing progenitors as suggested by dominance of the CD11b^+^/Lyve-1^+^ population as compared with minor CD3e^+^ and Ter-119^+^ subsets. The preference for expansion of lymphatics can be explained by their unique and essential role in removing stimulatory immune cells [86] and debris [87] that otherwise sustain chronic inflammation [88] and block tissue repair [89]. Therefore, increased lymphatics address the most pressing tumor need for regeneration that is less likely to be supported by local production of T-cells or erythrocytes plentifully supplied by blood vessels.

In summary, we discovered that the CSF-1/TLR4/IL-10 axis activated in BM cells *in vitro* and by tumors *in vivo* induced multipotent progenitors with the potential to develop into M2 myeloid, erythroid, T-cell lymphocytes, and lymphatic endothelial lineages. Maturation of these progenitors into discrete lineage-specific subsets was subsequently promoted in the tumor microenvironment that predominantly favored the expansion of M2-type myeloid-lymphatic cells. To our knowledge, this is the first evidence suggesting that tumors influence the cell composition of their environment. These findings advance our understanding of different stages in maturation of lymphatic progenitors in BM and tumors thus laying the foundation for approaches to counteract their generation for development of anti-metastatic therapies.

## Supporting information

S1 Fig Baseline expression of T-cell markers and Ter-119 in ex vivo bone marrow cells. Isolated bone marrow cells were stained with antibodies against T-cell markers and an erythroid marker Ter-119 followed by flow cytometry analysis. Representative flow cytometry histograms demonstrating expression of (A) CD3e, (B) CD4, (C) CD8, and (D) Ter-119 are shown (blue line). Percent of positive cells was determined based on cells stained with secondary antibody alone (black line).
(PDF)

S2 Fig Uniform expression of Lyve-1, CD3e and Ter-119 in GFP^+^ bone marrow cells differentiated with CSF-1 and LPS. Bone marrow cells isolated from mice with ubiquitous GFP expression were differentiated using standard protocol employing CSF-1 and LPS as described under Methods. (A) Ubiquitous GFP expression in 100% of cells was confirmed after staining with nuclear Hoechst dye by fluorescent and bright field microscopy. (B) Differentiated GFP-positive cells were stained for Lyve-1, CD3e and Ter-119 followed by flow cytometry analysis prior to adoptive transfer to tumor-bearing mice. Percent of positive cells (red line) was determined based on cells stained with secondary antibody alone (black line).
(PDF)

S1 Table Antibodies used for flow cytometry and immunohistochemistry. (PDF)

S2 Table RT-qPCR primers. (PDF)

## References

[1] Kesler CT, Liao S, Munn LL, Padera TP. Lymphatic vessels in health and disease. Wiley.Interdiscip.Rev.Syst.Biol.Med. 2013; 5: 111–124. doi: 10.1002/wsbm.1201 23209022 PMC3527689

[2] Alitalo K. The lymphatic vasculature in disease. Nat.Med. 2011; 17: 1371–1380. doi: 10.1038/nm.2545 22064427

[3] Ran S, Volk L, Hall K, Flister MJ. Lymphangiogenesis and lymphatic metastasis in breast cancer. Pathophysiology. 2009; 17: 229–251. doi: 10.1016/j.pathophys.2009.11.003 20036110 PMC2891887

[4] Brown M, Assen FP, Leithner A, Abe J, Schachner H, Asfour G, et al. Lymph node blood vessels provide exit routes for metastatic tumor cell dissemination in mice. Science. 2018; 359: 1408–1411. doi: 10.1126/science.aal3662 29567714

[5] Tan KW, Chong SZ, Angeli V. Inflammatory lymphangiogenesis: cellular mediators and functional implications. Angiogenesis. 2014; 17: 373–381. doi: 10.1007/s10456-014-9419-4 24449091

[6] Karpanen T, Egeblad M, Karkkainen MJ, Kubo H, Yla-Herttuala S, Jaattela M, et al. Vascular endothelial growth factor C promotes tumor lymphangiogenesis and intralymphatic tumor growth. Cancer Res. 2001; 61: 1786–1790. 11280723

[7] Achen MG, Williams RA, Baldwin ME, Lai P, Roufail S, Alitalo K, et al. The angiogenic and lymphangiogenic factor vascular endothelial growth factor-D exhibits a paracrine mode of action in cancer. Growth Factors. 2002; 20: 99–107. doi: 10.1080/08977190290031969 12148568

[8] He Y, Kozaki K, Karpanen T, Koshikawa K, Yla-Herttuala S, Takahashi T, et al. Suppression of tumor lymphangiogenesis and lymph node metastasis by blocking vascular endothelial growth factor receptor 3 signaling. J.Natl.Cancer Inst. 2002; 94: 819–825. doi: 10.1093/jnci/94.11.819 12048269

[9] Hall KL, Volk-Draper LD, Flister MJ, Ran S. New model of macrophage acquisition of the lymphatic endothelial phenotype. PLoS ONE. 2012; 7: e31794. doi: 10.1371/journal.pone.0031794 22396739 PMC3292559

[10] Ding M, Fu X, Tan H, Wang R, Chen Z, Ding S. The effect of vascular endothelial growth factor C expression in tumor-associated macrophages on lymphangiogenesis and lymphatic metastasis in breast cancer. Mol.Med.Report. 2012; 6: 1023–1029. doi: 10.3892/mmr.2012.1043 22923155

[11] Tawada M, Hayashi S, Ikegame Y, Nakashima S, Yoshida K. Possible involvement of tumor-producing VEGF-A in the recruitment of lymphatic endothelial progenitor cells from bone marrow. Oncol.Rep. 2014; 32: 2359–2364. doi: 10.3892/or.2014.3499 25242215

[12] Volk-Draper LD, Hall KL, Wilber AC, Ran S. Lymphatic endothelial progenitors originate from plastic myeloid cells activated by toll-like receptor-4. PLoS ONE. 2017; 12: e0179257. doi: 10.1371/journal.pone.0179257 28598999 PMC5466303

[13] Ran S, Volk-Draper L. Lymphatic Endothelial Cell Progenitors in the Tumor Microenvironment. Adv.Exp.Med.Biol. 2020; 1234: 87–105. doi: 10.1007/978-3-030-37184-5_7 32040857 PMC7263265

[14] Van’t Hull EF, Bron S, Henry L, Ifticene-Treboux A, Turrini R, Coukos G, et al. Bone marrow-derived cells are implicated as a source of lymphatic endothelial progenitors in human breast cancer. Oncoimmunology. 2014; 3: e29080. doi: 10.4161/onci.29080 25101222 PMC4121340

[15] Bogos K, Renyi-Vamos F, Dobos J, Kenessey I, Tovari J, Timar J, et al. High VEGFR-3-positive circulating lymphatic/vascular endothelial progenitor cell level is associated with poor prognosis in human small cell lung cancer. Clin.Cancer Res. 2009; 15: 1741–1746. doi: 10.1158/1078-0432.CCR-08-1372 19240177

[16] Volk-Draper L, Patel R, Bhattarai N, Yang J, Wilber A, DeNardo D, et al. Myeloid-Derived Lymphatic Endothelial Cell Progenitors Significantly Contribute to Lymphatic Metastasis in Clinical Breast Cancer. Am.J.Pathol. 2019; 189: 2269–2292. doi: 10.1016/j.ajpath.2019.07.006 31421071 PMC6892188

[17] Lee SJ, Park C, Lee JY, Kim S, Kwon PJ, Kim W, et al. Generation of pure lymphatic endothelial cells from human pluripotent stem cells and their therapeutic effects on wound repair. Sci.Rep. 2015; 5: 11019. doi: 10.1038/srep11019 26066093 PMC4464258

[18] Religa P, Cao R, Bjorndahl M, Zhou Z, Zhu Z, Cao Y. Presence of bone marrow-derived circulating progenitor endothelial cells in the newly formed lymphatic vessels. Blood. 2005; 106: 4184–4190. doi: 10.1182/blood-2005-01-0226 16141354

[19] Lee JY, Park C, Cho YP, Lee E, Kim H, Kim P, et al. Podoplanin-expressing cells derived from bone marrow play a crucial role in postnatal lymphatic neovascularization. Circulation. 2010; 122: 1413–1425. doi: 10.1161/CIRCULATIONAHA.110.941468 20855662 PMC2989430

[20] Bron S, Henry L, Faes-Van’t Hull E, Turrini R, Vanhecke D, Guex N, et al. TIE-2-expressing monocytes are lymphangiogenic and associate specifically with lymphatics of human breast cancer. Oncoimmunology. 2016; 5: e1073882. doi: 10.1080/2162402X.2015.1073882 27057438 PMC4801424

[21] Kurahara H, Shinchi H, Mataki Y, Maemura K, Noma H, Kubo F, et al. Significance of M2-polarized tumor-associated macrophage in pancreatic cancer. J.Surg.Res. 2011; 167: e211–e219. doi: 10.1016/j.jss.2009.05.026 19765725

[22] Dominguez-Soto A, Sierra-Filardi E, Puig-Kroger A, Perez-Maceda B, Gomez-Aguado F, Corcuera MT, et al. Dendritic cell-specific ICAM-3-grabbing nonintegrin expression on M2-polarized and tumor-associated macrophages is macrophage-CSF dependent and enhanced by tumor-derived IL-6 and IL-10. J.Immunol. 2011; 186: 2192–2200. doi: 10.4049/jimmunol.1000475 21239715

[23] Schmieder A, Schledzewski K, Michel J, Tuckermann JP, Tome L, Sticht C, et al. Synergistic activation by p38MAPK and glucocorticoid signaling mediates induction of M2-like tumor-associated macrophages expressing the novel CD20 homolog MS4A8 A. Int.J.Cancer. 2011; 129: 122–132. 20824698 10.1002/ijc.25657

[24] Ho VW, Sly LM. Derivation and characterization of murine alternatively activated (M2) macrophages. Methods Mol.Biol. 2009; 531: 173–185. doi: 10.1007/978-1-59745-396-7_12 19347318

[25] Espinosa Gonzalez M., Volk-Draper L, Bhattarai N, Wilber A, Ran S. Th2 Cytokines IL-4, IL-13, and IL-10 Promote Differentiation of Pro-Lymphatic Progenitors Derived from Bone Marrow Myeloid Precursors. Stem Cells Dev. 2022; 31: 322–333. doi: 10.1089/scd.2022.0004 35442077 PMC9232236

[26] Kang YJ, Yang SJ, Park G, Cho B, Min CK, Kim TY, et al. A novel function of interleukin-10 promoting self-renewal of hematopoietic stem cells. Stem Cells. 2007; 25: 1814–1822. doi: 10.1634/stemcells.2007-0002 17464085

[27] Rieser C, Ramoner R, Bock G, Deo YM, Holtl L, Bartsch G, et al. Human monocyte-derived dendritic cells produce macrophage colony-stimulating factor: enhancement of c-fms expression by interleukin-10. Eur.J.Immunol. 1998; 28: 2283–2288. doi: 10.1002/(SICI)1521-4141(199808)28:08&lt;2283::AID-IMMU2283&gt;3.0.CO;2-X 9710206

[28] Heine G, Drozdenko G, Grun JR, Chang HD, Radbruch A, Worm M. Autocrine IL-10 promotes human B-cell differentiation into IgM- or IgG-secreting plasmablasts. Eur.J.Immunol. 2014; 44: 1615–1621. doi: 10.1002/eji.201343822 24643722

[29] Krishnamurthy P, Thal M, Verma S, Hoxha E, Lambers E, Ramirez V, et al. Interleukin-10 deficiency impairs bone marrow-derived endothelial progenitor cell survival and function in ischemic myocardium. Circ.Res. 2011; 109: 1280–1289. doi: 10.1161/CIRCRESAHA.111.248369 21959218 PMC3235675

[30] Tang H, Guo Z, Zhang M, Wang J, Chen G, Cao X. Endothelial stroma programs hematopoietic stem cells to differentiate into regulatory dendritic cells through IL-10. Blood. 2006; 108: 1189–1197. doi: 10.1182/blood-2006-01-007187 16627758

[31] Hashimoto S, Yamada M, Motoyoshi K, Akagawa KS. Enhancement of macrophage colony-stimulating factor-induced growth and differentiation of human monocytes by interleukin-10. Blood. 1997; 89: 315–321. 8978307

[32] Allavena P, Piemonti L, Longoni D, Bernasconi S, Stoppacciaro A, Ruco L, et al. IL-10 prevents the differentiation of monocytes to dendritic cells but promotes their maturation to macrophages. Eur.J.Immunol. 1998; 28: 359–369. doi: 10.1002/(SICI)1521-4141(199801)28:01&lt;359::AID-IMMU359&gt;3.0.CO;2-4 9485215

[33] Meng L, Almeida LN, Clauder AK, Lindemann T, Luther J, Link C, et al. Bone Marrow Plasma Cells Modulate Local Myeloid-Lineage Differentiation via IL-10. Front Immunol. 2019; 10: 1183. doi: 10.3389/fimmu.2019.01183 31214168 PMC6555095

[34] Murai M, Turovskaya O, Kim G, Madan R, Karp CL, Cheroutre H, et al. Interleukin 10 acts on regulatory T cells to maintain expression of the transcription factor Foxp3 and suppressive function in mice with colitis. Nat.Immunol. 2009; 10: 1178–1184. doi: 10.1038/ni.1791 19783988 PMC2898179

[35] Cui K, Chen Y, Zhong H, Wang N, Zhou L, Jiang F. Transplantation of IL-10-Overexpressing Bone Marrow-Derived Mesenchymal Stem Cells Ameliorates Diabetic-Induced Impaired Fracture Healing in Mice. Cell Mol.Bioeng. 2020; 13: 155–163. doi: 10.1007/s12195-019-00608-w 32175028 PMC7048895

[36] Chen E, Liu G, Zhou X, Zhang W, Wang C, Hu D, et al. Concentration-dependent, dual roles of IL-10 in the osteogenesis of human BMSCs via P38/MAPK and NF-kappaB signaling pathways. FASEB J. 2018; 32: 4917–4929. 29630408 10.1096/fj.201701256RRR

[37] Behrendt P, Feldheim M, Preusse-Prange A, Weitkamp JT, Haake M, Eglin D, et al. Chondrogenic potential of IL-10 in mechanically injured cartilage and cellularized collagen ACI grafts. Osteoarthritis.Cartilage. 2018; 26: 264–275. doi: 10.1016/j.joca.2017.11.007 29169959

[38] Jagielski M, Wolf J, Marzahn U, Volker A, Lemke M, Meier C, et al. The influence of IL-10 and TNFalpha on chondrogenesis of human mesenchymal stromal cells in three-dimensional cultures. Int.J.Mol.Sci. 2014; 15: 15821–15844. 25207597 10.3390/ijms150915821 PMC4200793

[39] Rajbhandari P, Thomas BJ, Feng AC, Hong C, Wang J, Vergnes L, et al. IL-10 Signaling Remodels Adipose Chromatin Architecture to Limit Thermogenesis and Energy Expenditure. Cell. 2018; 172: 218–233. doi: 10.1016/j.cell.2017.11.019 29249357 PMC5766418

[40] Jenkins BR, Blaseg NA, Grifka-Walk HM, Deuling B, Swain SD, Campbell EL, et al. Loss of interleukin-10 receptor disrupts intestinal epithelial cell proliferation and skews differentiation towards the goblet cell fate. FASEB J. 2021; 35: e21551. doi: 10.1096/fj.202002369R 34042222

[41] Yang J, Jiang Z, Fitzgerald DC, Ma C, Yu S, Li H, et al. Adult neural stem cells expressing IL-10 confer potent immunomodulation and remyelination in experimental autoimmune encephalitis. J.Clin.Invest. 2009; 119: 3678–3691. doi: 10.1172/JCI37914 19884657 PMC2786785

[42] Antoniv TT, Ivashkiv LB. Dysregulation of interleukin-10-dependent gene expression in rheumatoid arthritis synovial macrophages. Arthritis Rheum. 2006; 54: 2711–2721. doi: 10.1002/art.22055 16947381

[43] Diefenhardt P, Nosko A, Kluger MA, Richter JV, Wegscheid C, Kobayashi Y, et al. IL-10 Receptor Signaling Empowers Regulatory T Cells to Control Th17 Responses and Protect from GN. J.Am.Soc.Nephrol. 2018; 29: 1825–1837. doi: 10.1681/ASN.2017091044 29866800 PMC6050938

[44] Nitahara-Kasahara Y, Kuraoka M, Oda Y, Hayashita-Kinoh H, Takeda S, Okada T. Enhanced cell survival and therapeutic benefits of IL-10-expressing multipotent mesenchymal stromal cells for muscular dystrophy. Stem Cell Res.Ther. 2021; 12: 105. doi: 10.1186/s13287-021-02168-1 33541428 PMC7860619

[45] Gordon S, Martinez FO. Alternative activation of macrophages: mechanism and functions. Immunity. 2010; 32: 593–604. doi: 10.1016/j.immuni.2010.05.007 20510870

[46] Umemura N, Saio M, Suwa T, Kitoh Y, Bai J, Nonaka K, et al. Tumor-infiltrating myeloid-derived suppressor cells are pleiotropic-inflamed monocytes/macrophages that bear M1- and M2-type characteristics. J.Leukoc.Biol. 2008; 83: 1136–1144. doi: 10.1189/jlb.0907611 18285406

[47] Bronte V, Brandau S, Chen SH, Colombo MP, Frey AB, Greten TF, et al. Recommendations for myeloid-derived suppressor cell nomenclature and characterization standards. Nat.Commun. 2016; 7: 12150. doi: 10.1038/ncomms12150 27381735 PMC4935811

[48] Daassi D, Hamada M, Jeon H, Imamura Y, Nhu Tran MT, Takahashi S. Differential expression patterns of MafB and c-Maf in macrophages in vivo and in vitro. Biochem.Biophys.Res.Commun. 2016; 473: 118–124. doi: 10.1016/j.bbrc.2016.03.063 26996125

[49] Baker RG, Hayden MS, Ghosh S. NF-kappaB, inflammation, and metabolic disease. Cell Metab. 2011; 13: 11–22. 21195345 10.1016/j.cmet.2010.12.008 PMC3040418

[50] Bhattacharjee A, Shukla M, Yakubenko VP, Mulya A, Kundu S, Cathcart MK. IL-4 and IL-13 employ discrete signaling pathways for target gene expression in alternatively activated monocytes/macrophages. Free Radic.Biol.Med. 2013; 54: 1–16. doi: 10.1016/j.freeradbiomed.2012.10.553 23124025 PMC3534796

[51] Huang H, Zheng Y, Li L, Shi W, Zhang R, Liu H, et al. The roles of post-translational modifications and coactivators of STAT6 signaling in tumor growth and progression. Future.Med.Chem. 2020; 12: 1945–1960. doi: 10.4155/fmc-2020-0224 32779479

[52] Romagnani P, Annunziato F, Liotta F, Lazzeri E, Mazzinghi B, Frosali F, et al. CD14+CD34low cells with stem cell phenotypic and functional features are the major source of circulating endothelial progenitors. Circ.Res. 2005; 97: 314–322. doi: 10.1161/01.RES.0000177670.72216.9b 16020753

[53] Ye C, Bai L, Yan ZQ, Wang YH, Jiang ZL. Shear stress and vascular smooth muscle cells promote endothelial differentiation of endothelial progenitor cells via activation of Akt. Clin.Biomech. 2007; 23: S118–S124. doi: 10.1016/j.clinbiomech.2007.08.018 17928113

[54] Sauer H, Bekhite MM, Hescheler J, Wartenberg M. Redox control of angiogenic factors and CD31-positive vessel-like structures in mouse embryonic stem cells after direct current electrical field stimulation. Exp.Cell Res. 2005; 304: 380–390. doi: 10.1016/j.yexcr.2004.11.026 15748885

[55] Lin G, Finger E, Gutierrez-Ramos JC. Expression of CD34 in endothelial cells, hematopoietic progenitors and nervous cells in fetal and adult mouse tissues. Eur.J.Immunol. 1995; 25: 1508–1516. doi: 10.1002/eji.1830250606 7542195

[56] Venable A, Mitalipova M, Lyons I, Jones K, Shin S, Pierce M, et al. Lectin binding profiles of SSEA-4 enriched, pluripotent human embryonic stem cell surfaces. BMC Dev.Biol. 2005; 5: 15. doi: 10.1186/1471-213X-5-15 16033656 PMC1182361

[57] Adini A, Adini I, Ghosh K, Benny O, Pravda E, Hu R, et al. The stem cell marker prominin-1/CD133 interacts with vascular endothelial growth factor and potentiates its action. Angiogenesis. 2013; 16: 405–416. doi: 10.1007/s10456-012-9323-8 23150059

[58] Deshpande S, Bosbach B, Yozgat Y, Park CY, Moore MA, Besmer P. KIT receptor gain-of-function in hematopoiesis enhances stem cell self-renewal and promotes progenitor cell expansion. Stem Cells. 2013; 31: 1683–1695. doi: 10.1002/stem.1419 23681919 PMC3775897

[59] Xiao Q, Zeng L, Zhang Z, Margariti A, Ali ZA, Channon KM, et al. Sca-1+ progenitors derived from embryonic stem cells differentiate into endothelial cells capable of vascular repair after arterial injury. Arterioscler.Thromb.Vasc.Biol. 2006; 26: 2244–2251. doi: 10.1161/01.ATV.0000240251.50215.50 16902164

[60] Rivas JR, Liu Y, Alhakeem SS, Eckenrode JM, Marti F, Collard JP, et al. Interleukin-10 suppression enhances T-cell antitumor immunity and responses to checkpoint blockade in chronic lymphocytic leukemia. Leukemia. 2021; 35: 3188–3200. doi: 10.1038/s41375-021-01217-1 33731852 PMC8446094

[61] Ran S, Wilber A. Novel role of immature myeloid cells in formation of new lymphatic vessels associated with inflammation and tumors. J.Leukoc.Biol. 2017; 102: 253–263. doi: 10.1189/jlb.1MR1016-434RR 28408396 PMC5505745

[62] Martinez FO, Sica A, Mantovani A, Locati M. Macrophage activation and polarization. Front Biosci. 2008; 13: 453–461. doi: 10.2741/2692 17981560

[63] Makita N, Hizukuri Y, Yamashiro K, Murakawa M, Hayashi Y. IL-10 enhances the phenotype of M2 macrophages induced by IL-4 and confers the ability to increase eosinophil migration. Int.Immunol. 2015; 27: 131–141. doi: 10.1093/intimm/dxu090 25267883

[64] Ng TH, Britton GJ, Hill EV, Verhagen J, Burton BR, Wraith DC. Regulation of adaptive immunity; the role of interleukin-10. Front Immunol. 2013; 4: 129. doi: 10.3389/fimmu.2013.00129 23755052 PMC3668291

[65] Conrad C, Niess H, Huss R, Huber S, von L, I, Nelson PJ, et al. Multipotent mesenchymal stem cells acquire a lymphendothelial phenotype and enhance lymphatic regeneration in vivo. Circulation. 2009; 119: 281–289. doi: 10.1161/CIRCULATIONAHA.108.793208 19118255

[66] Kobayashi T, Matsuoka K, Sheikh SZ, Russo SM, Mishima Y, Collins C, et al. IL-10 regulates Il12b expression via histone deacetylation: implications for intestinal macrophage homeostasis. J.Immunol. 2012; 189: 1792–1799. doi: 10.4049/jimmunol.1200042 22786766 PMC3411910

[67] Lewkowicz N, Mycko MP, Przygodzka P, Cwiklinska H, Cichalewska M, Matysiak M, et al. Induction of human IL-10-producing neutrophils by LPS-stimulated Treg cells and IL-10. Mucosal.Immunol. 2016; 9: 364–378. doi: 10.1038/mi.2015.66 26220165

[68] Lang R, Patel D, Morris JJ, Rutschman RL, Murray PJ. Shaping gene expression in activated and resting primary macrophages by IL-10. J.Immunol. 2002; 169: 2253–2263. doi: 10.4049/jimmunol.169.5.2253 12193690

[69] Cardon T, Ozcan B, Aboulouard S, Kobeissy F, Duhamel M, Rodet F, et al. Epigenetic Studies Revealed a Ghost Proteome in PC1/3 KD Macrophages under Antitumoral Resistance Induced by IL-10. ACS Omega. 2020; 5: 27774–27782. doi: 10.1021/acsomega.0c02530 33163760 PMC7643081

[70] Mercer EM, Lin YC, Benner C, Jhunjhunwala S, Dutkowski J, Flores M, et al. Multilineage priming of enhancer repertoires precedes commitment to the B and myeloid cell lineages in hematopoietic progenitors. Immunity. 2011; 35: 413–425. doi: 10.1016/j.immuni.2011.06.013 21903424 PMC3183365

[71] Tanaka N, Hoshino Y, Gold J, Hoshino S, Martiniuk F, Kurata T, et al. Interleukin-10 induces inhibitory C/EBPbeta through STAT-3 and represses HIV-1 transcription in macrophages. Am.J.Respir.Cell Mol.Biol. 2005; 33: 406–411. doi: 10.1165/rcmb.2005-0140OC 16014896 PMC2715348

[72] Parmar N, Chandrakar P, Kar S. Leishmania donovani Subverts Host Immune Response by Epigenetic Reprogramming of Macrophage M(Lipopolysaccharides + IFN-gamma)/M(IL-10) Polarization. J.Immunol. 2020; 204: 2762–2778. 32277055 10.4049/jimmunol.1900251

[73] Mancheno-Corvo P, Lopez-Santalla M, Menta R, DelaRosa O, Mulero F, Del RB, et al. Intralymphatic Administration of Adipose Mesenchymal Stem Cells Reduces the Severity of Collagen-Induced Experimental Arthritis. Front Immunol. 2017; 8: 462. doi: 10.3389/fimmu.2017.00462 28484460 PMC5399019

[74] Salven P, Mustjoki S, Alitalo R, Alitalo K, Rafii S. VEGFR-3 and CD133 identify a population of CD34+ lymphatic/vascular endothelial precursor cells. Blood. 2003; 101: 168–172. doi: 10.1182/blood-2002-03-0755 12393704

[75] Loukovaara S, Gucciardo E, Repo P, Vihinen H, Lohi J, Jokitalo E, et al. Indications of lymphatic endothelial differentiation and endothelial progenitor cell activation in the pathology of proliferative diabetic retinopathy. Acta Ophthalmol. 2015; 93: 512–523. doi: 10.1111/aos.12741 25899460

[76] Aranguren XL, McCue JD, Hendrickx B, Zhu XH, Du F, Chen E, et al. Multipotent adult progenitor cells sustain function of ischemic limbs in mice. J.Clin.Invest. 2008; 118: 505–514. doi: 10.1172/JCI31153 18172550 PMC2157560

[77] Aranguren XL, Luttun A, Clavel C, Moreno C, Abizanda G, Barajas MA, et al. In vitro and in vivo arterial differentiation of human multipotent adult progenitor cells. Blood. 2007; 109: 2634–2642. doi: 10.1182/blood-2006-06-030411 17090652

[78] Kodama H, Inoue T, Watanabe R, Yasutomi D, Kawakami Y, Ogawa S, et al. Neurogenic potential of progenitors derived from human circulating CD14+ monocytes. Immunol.Cell Biol. 2006; 84: 209–217. doi: 10.1111/j.1440-1711.2006.01424.x 16519739

[79] Kuwana M, Okazaki Y, Kodama H, Izumi K, Yasuoka H, Ogawa Y, et al. Human circulating CD14+ monocytes as a source of progenitors that exhibit mesenchymal cell differentiation. J.Leukoc.Biol. 2003; 74: 833–845. doi: 10.1189/jlb.0403170 12960274

[80] Maertens L, Erpicum C, Detry B, Blacher S, Lenoir B, Carnet O, et al. Bone marrow-derived mesenchymal stem cells drive lymphangiogenesis. PLoS ONE. 2014; 9: e106976. doi: 10.1371/journal.pone.0106976 25222747 PMC4164522

[81] Cortez-Retamozo V, Etzrodt M, Newton A, Rauch PJ, Chudnovskiy A, Berger C, et al. Origins of tumor-associated macrophages and neutrophils. Proc.Natl.Acad.Sci.U.S.A. 2012; 109: 2491–2496. doi: 10.1073/pnas.1113744109 22308361 PMC3289379

[82] Shand FH, Ueha S, Otsuji M, Koid SS, Shichino S, Tsukui T, et al. Tracking of intertissue migration reveals the origins of tumor-infiltrating monocytes. Proc.Natl.Acad.Sci.U.S.A. 2014; 111: 7771–7776. doi: 10.1073/pnas.1402914111 24825888 PMC4040600

[83] Wu C, Ning H, Liu M, Lin J, Luo S, Zhu W, et al. Spleen mediates a distinct hematopoietic progenitor response supporting tumor-promoting myelopoiesis. J.Clin.Invest. 2018; 128: 3425–3438. doi: 10.1172/JCI97973 29771686 PMC6063469

[84] Swirski FK, Nahrendorf M, Etzrodt M, Wildgruber M, Cortez-Retamozo V, Panizzi P, et al. Identification of splenic reservoir monocytes and their deployment to inflammatory sites. Science. 2009; 325: 612–616. doi: 10.1126/science.1175202 19644120 PMC2803111

[85] Szilvassy SJ, Bass MJ, Van ZG, Grimes B. Organ-selective homing defines engraftment kinetics of murine hematopoietic stem cells and is compromised by Ex vivo expansion. Blood. 1999; 93: 1557–1566. 10029584

[86] Ravaud C, Ved N, Jackson DG, Vieira JM, Riley PR. Lymphatic Clearance of Immune Cells in Cardiovascular Disease. Cells. 2021; 10: 2594. doi: 10.3390/cells10102594 34685572 PMC8533855

[87] Vieira JM, Norman S, Villa Del CC, Cahill TJ, Barnette DN, Gunadasa-Rohling M, et al. The cardiac lymphatic system stimulates resolution of inflammation following myocardial infarction. J.Clin.Invest. 2018; 128: 3402–3412. doi: 10.1172/JCI97192 29985167 PMC6063482

[88] Guo R, Zhou Q, Proulx ST, Wood R, Ji RC, Ritchlin CT, et al. Inhibition of lymphangiogenesis and lymphatic drainage via vascular endothelial growth factor receptor 3 blockade increases the severity of inflammation in a mouse model of chronic inflammatory arthritis. Arthritis Rheum. 2009; 60: 2666–2676. doi: 10.1002/art.24764 19714652 PMC2810533

[89] Church JS, Kigerl KA, Lerch JK, Popovich PG, McTigue DM. TLR4 Deficiency Impairs Oligodendrocyte Formation in the Injured Spinal Cord. J.Neurosci. 2016; 36: 6352–6364. doi: 10.1523/JNEUROSCI.0353-16.2016 27277810 PMC4899531

