# Supplementary figures and images for: Tumor microenvironment restricts IL-10 induced multipotent progenitors to myeloid-lymphatic phenotype
Source: PLoS One. 2024 Apr 19;19(4):e0298465. doi: 10.1371/journal.pone.0298465 (PMC11029653; doi:10.1371/journal.pone.0298465)

— Sec. ctrl

— Ex vivo

**CD3e**

**CD4**

**CD8**

**Ter-119**

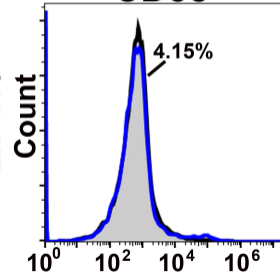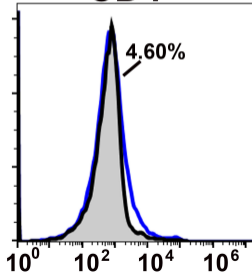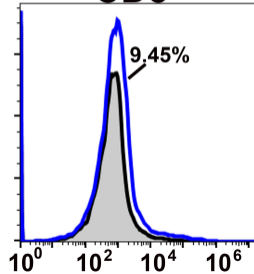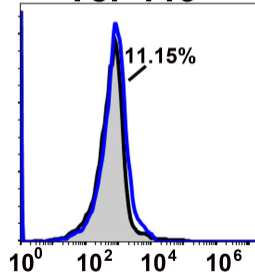

Supplement: S1 Fig — Isolated bone marrow cells were stained with antibodies against T-cell markers and an erythroid marker Ter-119 followed by flow cytometry analysis. Representative flow cytometry histograms demonstrating expression of (A) CD3e, (B) CD4, (C) CD8, and (D) Ter-119 are shown (blue line). Percent of positive cells was determined based on cells stained with secondary antibody alone (black line). (PDF) [file pone.0298465.s001.pdf]

**A**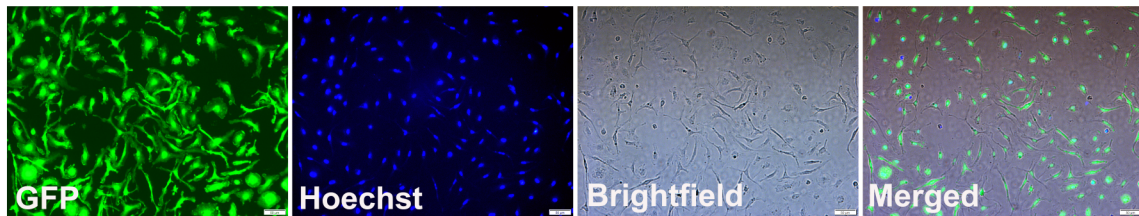**B**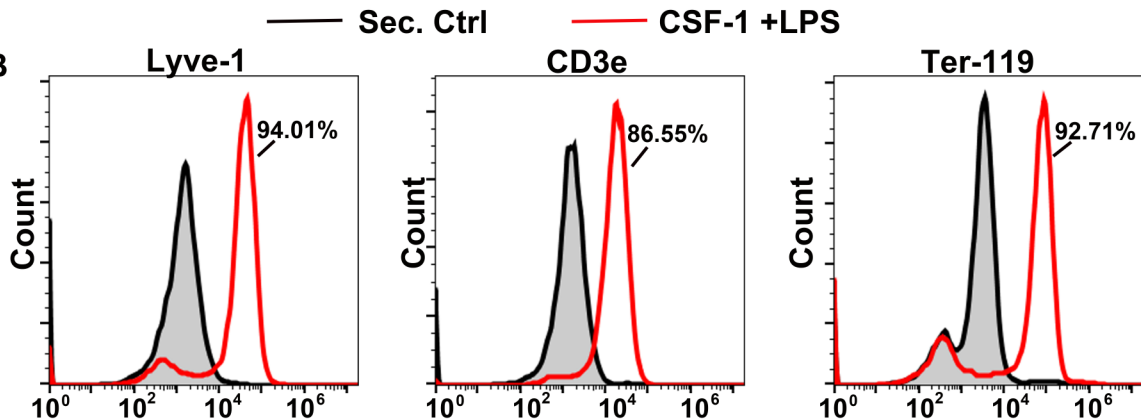

Supplement: S2 Fig — Bone marrow cells isolated from mice with ubiquitous GFP expression were differentiated using standard protocol employing CSF-1 and LPS as described under Methods. (A) Ubiquitous GFP expression in 100% of cells was confirmed after staining with nuclear Hoechst dye by fluorescent and bright field microscopy. (B) Differentiated GFP-positive cells were stained for Lyve-1, CD3e and Ter-119 followed by flow cytometry analysis prior to adoptive transfer to tumor-bearing mice. Percent of positive cells (red line) was determined based on cells stained with secondary antibody alone (black line). (PDF) [file pone.0298465.s002.pdf]
