# Supplementary material for: Tumor microenvironment restricts IL-10 induced multipotent progenitors to myeloid-lymphatic phenotype
Source: PLoS One. 2024 Apr 19;19(4):e0298465. doi: 10.1371/journal.pone.0298465 (PMC11029653; doi:10.1371/journal.pone.0298465)
Supplement: S2 Table — (PDF) [file pone.0298465.s004.pdf]

Supplemental Table S2. RT-qPCR primers

*Gene**	Product Size (bp)	Forward Sequence (5' → 3')	Reverse Sequence (5' → 3')
Actb	153	GGCTGTATTCCCCTCCATCG	CCAGTTGGTAACAATGCCATGT
Cd3	125	ATGCGGTGGAACACTTTCTGG	GCACGTCAACTCTACACTGGT
Cd4	295	CCAACAGCGCCAGGCA	AGTGTCTGAAACGCAGAGGG
Cd8	165	TTCTGTCGTGCCAGTCCTTC	TGGGACATTTGCAAACACGC
Cd19	163	GGAGGCAATGTTGTGCTGC	ACAATCACTAGCAAGATGCCC
Cd23	165	CCCTTCTCTCCCATTCCTTC	AGACAAAGGGGGCTTTTGAT
CD31	196	TGCACCCATCACTTACCACC	CTTCATCCACCGGGGCTATC
Cd34	156	AAGGCTGGGTGAAGACCCTTA	TGAATGGCCGTTTCTGGAAGT
Cd41	132	TTCTTGGGTCCTAGTGCTGTT	CGCTTCCATGTTTGTCCTTATGA
Cd63	138	AGAGACCAGGTGAAGTCAGAG	AGTCTGTGTAGTTAGAAGCTCCA
Cd163	108	GGTGGACACAGAATGGTTCTTC	CCAGGAGCGTTAGTGACAGC
Cd204	166	AGTGCTGTCTTCTTTACCAGCA	CTGAAGGGAGGGGCCATTTT
Cd206	88	GAGGGAAGCGAGAGATTATGGA	GCCTGATGCCAGGGTAAAGCA
Cd209	110	GTTTGTTCTGTGCTGCTGGTT	GCCTTCAACTGGGTCAGTTCT
Cd226	159	CAGTGCTTGCTATCAGTGGC	TTGGGCGTAACTCTCCTTGC
cMaf	104	AGCAAGGAGGAGGTGATCCG	TGTCTCTGCTGCACCCTCTTG
Colec12	95	GCAGTGAGAGACACTGGTACG	GAAGGACTGCACCTCCTCTTC
Gata1	70	CAGAACCGGCCTCTCATCC	TAGTGCATTGGGTGCCTGC
Hemgn	74	CAAAAGCCCTCCCCCAATCA	TTCTGGTGTGACTGACGTGG
Ifl16	272	AAAGGAGCCTGCTAAGGAAGA	CGTTCACATCAGAGACACAGGA
Ifng	136	GAGGTCAACAACCCACAGGT	GGGACAATCTCTTCCCCACC
Il1a	251	CGCTTGAGTCGGCAAAGAAA	CTGATACTGTCACCCGGCTC
Il1b	88	GCAACTGTTCCTGAACTCAACT	ATCTTTTGGGGTCCGTCAACT
Il2r	159	GGACAGAGTGTTCACTACGA	CCTCACTAGCCAGAAATCGG
Il4	101	GGTCTCAACCCCCAGCTAGT	GCCGATGATCTCTCTCAAGTGAT
Il4r	119	TCTGTGGGCTGTCTGATTTT	GCTATCCAGGAACCACTCAC
Il6	131	TTCTTGGGACTGATGCTGGT	TGTGTAATTAAGCCTCCGAC
Il10	277	CCCAGTCGGCCAGAGC	GCGCCTCAGCCGCAT
Il10r	100	CAAACAGTACGGAAACTCAACCT	GGTGATACAGATCCAGGGTGAAC
Il12a	76	AGACATCACACGGGACCAAAC	CCAGGCAACTCTCGTTCTTGT
Il12ra	148	CGCGTGGGAGTCAGAGTG	AAAGCTAAAGCGCTGGGGAT
Il13	116	CCTGGCTCTTGCTTGCCTT	GGTCTTGTGTGATGTTGCTCA
Il13r	119	CAACAGGATAAGAAAATTGC	AGGGCTAGGCTTCTCACTTT
Il18	168	GACTCTTGCGTCAACTTCAAGG	CAGGCTGTCTTTTGTCAACGA
Irf8	182	CGGGGCTGATCTGGGAAAAT	CACAGCGTAACCTCGTCTTC
Irf9	116	TCAGGCCCTGCCCATTTCTT	TGCAGCGTACTTTGCCTGA
Kynu	193	TCGAGGAGGAAGGAGACTCG	CAGCAGGCAAAGTCAACACC

Ly6c	217	ACTGTGCCTGCAACCTTGT	TGCAGAATCCATCAGAGGCG
Ly6g	162	GACTTCCTGCAACACAACTACC	ACAGCATTACCAGTGATCTCAGT
Mafb	178	TTCGACCTTCTCAAGTTCGACG	TCGAGATGGGTCTTCGGTTCA
Nfkb1	164	ACACGAGGCTACAACTCTGC	GGTACCCCCAGAGACCTCAT
Pdpn	158	ACCGTGCCAGTGTTGTTCTG	AGCACCTGTGGTTGTTATTTTGT
Stab1	146	GGCAGACGGTACGGTCTAAAC	AGCGGCAGTCCAGAAGTATCT
Stab2	227	GCTGAGTGTGAGGTGTGGAA	TCTGTAGTGGGCATCTGGGA
Stat3	142	TACCTCTACCCCGACATTCCC	CATCAATGAATGGTGTCACACAGA
Stat6	189	TGGTCCTGGTCCAAGTGAGG	ATTTCCACCAGGCTTTCACA
Tgfβ1	114	CTCCCGTGGCTTCTAGTGC	GCCTTAGTTTGGACAGGATCTG
Tie2	66	CGGCCAGGTACATAGGAGGAA	TCACATCTCCGAACAATCAGC
Tnfa	102	CCACCACGCTCTTCTGTCTAC	AGGGTCTGGGCCATAGAACT
Tnfr1β	65	ACACCCTACAAACCGGAACC	AGCCTTCCTGTCATAGTATTCCT
Vdr	94	GGGCTTCCACTTCAACGCTA	ATTGAAGGGGCAGGTGAACA
Vegfr1	162	CTCAGACAAGTCAAACCTGGAG	GGGAACTTCATCTGGGTCCATAA
Vegfr2	132	TTTGGCAAATACAACCCTTCAGA	GCAGAAGATACTGTCACCACC
Vegfr3	194	CGGGCTACCTGTCCATCATC	TGTCACAGCTGCTGCCTTTA

[*] Primers were designed based on mouse CDS of targets found in NCBI database and validated using mouse universal cDNA.
